# 2026 Update on the Management of Diffuse Large B‐Cell Lymphoma

**DOI:** 10.1002/ajh.70229

**Published:** 2026-02-07

**Authors:** Elise A. Chong, Emily B. Tomasulo, Stefan K. Barta

**Affiliations:** ^1^ Lymphoma Program, Abramson Cancer Center Perelman School of Medicine at the University of Pennsylvania Philadelphia Pennsylvania USA; ^2^ Division of Hematology/Oncology Hospital of the University of Pennsylvania Philadelphia Pennsylvania USA

**Keywords:** CART cell therapy, chemotherapy, DLBCL, immunotherapy, lymphoma

## Abstract

Diffuse large B‐cell lymphoma (DLBCL) is the most common type of NHL in the Western Hemisphere. It comprises a heterogenous group of lymphomas, with different biology and clinical prognoses. R‐CHP remains the backbone of therapy, and frontline therapeutic options in fit patients are pola‐R‐CHP and R‐CHOP, whereas elderly or frail/unfit patients may be treated with R‐mini‐CHOP or palliation. Frontline trials aim to improve outcomes for patients with high‐risk disease utilizing R‐CHOP + novel agents, CAR‐T, and bispecific antibodies. Trials in the elderly/unfit population are minimizing and omitting chemotherapy. Risk‐adapted approaches targeting cell of origin (COO) and utilizing interim PET imaging or ctDNA to guide therapy escalation or deescalation remain under investigation. Second line therapy curative‐intent approaches include CAR‐T or autologous stem cell transplantation, depending upon timing of disease progression after first‐line therapy. In the relapsed/refractory setting, there has been a rapid growth in the therapeutic armamentarium, including bispecific antibody combinations with chemotherapy, bispecific antibodies with antibody‐drug conjugates, and brentuximab vedotin + lenalidomide + rituximab. Multiple novel trials are further advancing the field away from chemotherapy including targeted therapy‐antibody combinations, new bispecific antibodies and bispecific antibody combinations, immunomodulatory agents, and cellular therapy. In this review, we summarize recent data and discuss ongoing efforts to improve the management of DLBCL.

## 1 Introduction

Non‐Hodgkin lymphoma (NHL) is the most common hematological malignancy [1]. Among non‐Hodgkin lymphomas, large B‐cell lymphomas comprise a heterogeneous group of 18 distinct types of large B‐cell lymphomas [2]. Diffuse large B‐cell lymphoma NOS (DLBCL not otherwise specified) is the most frequently diagnosed NHL subtype and the most common type of large B‐cell lymphoma, representing about 30%–40% of adult lymphomas in the Western Hemisphere [2, 3] with an estimated 5.2 cases per 100 000 persons cases in the US in 2023 and over 20 000 new diagnoses annually. In the US from 2018 to 2022, the average age at diagnosis was 67 years with increased incidence in the elderly. There is a slight male predominance, and Hispanic and White populations have the highest rates compared to others, including Asian, and Black populations [3] (SEER accessed 12/17/2025, 12/18/2025). The etiology of DLBCL is typically unknown, but risk factors include viral, environmental and occupational exposures, genetic features (GWAS studies) involving genes in immune function pathways, immunosuppression, and chronic inflammatory states [2]. Most patients present with rapid growth of lymphadenopathy, although any site may be affected. In fact, 30%–40% of patients may present with solely extranodal disease [2]. B symptoms, including fever, night sweats, and weight loss or other symptoms resulting from the location of lymphomatous involvement may or may not be present. Diagnosis is typically made upon biopsy of an abnormal lymph node or extranodal lesion, which shows partial or total effacement of normal tissue architecture by medium‐sized to large lymphoid cells with a diffuse or vaguely nodular growth pattern that express CD45 and pan B‐cell markers (CD19, CD20, CD22, CD79a, and/or PAX5) and a mature B‐cell phenotype [2].

The clinical course of DLBCL tends to be aggressive, with survival typically measured in weeks to months without therapy. Although first line chemoimmunotherapy may cure most patients, outcomes for patients with relapsed DLBCL are poor, and those patients with primary refractory DLBCL have a significantly poorer prognosis, even despite multiple new additions to the therapeutic armamentarium. Five‐year survival in the US in 2015–2021 was approximately 65%, with poorer 5‐year survival of 56% in older patients ages 65 and older [3].

In this clinical update, we summarize recent data and discuss new milestones and advances in the management of frontline and relapsed/refractory DLBCL.

## 2 Classification

The diagnosis of DLBCL NOS is one of exclusion: large B‐cell lymphomas (LBCL) that do not meet criteria for one of the other specific types of large B‐cell lymphoma are classified as DLBCL NOS [2]. Although a detailed comparison of changes in the classification of LBCLs between WHO 4th Edition and WHO 2024 5th Edition is beyond the scope of this review, it is worth noting a few changes. High‐grade B‐cell lymphoma (HGBCL) with MYC and BCL2 and/or BCL6 rearrangements is now limited to either DLBCL or HGBCL with MYC & BCL2 rearrangements, whereas LBCL with MYC and BCL6 rearrangements is typically classified as DLBCL NOS and occasionally HGBCL NOS. Burkitt‐like lymphoma w/11q aberration is now recognized as different from Burkitt lymphoma (BL) and is now termed HGBCL with 11q aberration. Additionally, extranodal lymphomas of immune privileged sites are now considered one entity; this includes primary CNS, vitreoretinal, and testicular LBCL. Finally, it should also be noted that two different classifications were published nearly simultaneously. The 5th Edition WHO changes were published in Leukemia and the International Consensus Classification was released in Blood, both in 2022 [4, 5]. For LBCL, there are, fortunately, relatively small differences [6, 7]. This update focuses on DLBCL NOS, with brief inclusion of diffuse large B‐cell lymphoma/high grade B‐cell lymphoma with MYC and BCL2 rearrangements.

Multidisciplinary efforts have identified unique DLBCL subtypes by either cell of origin (COO) or molecular characteristics. These classification systems are now routinely used to identify subsets of patients with high‐risk disease and poorer outcomes to up‐front standard R‐CHOP therapy. Currently, DLBCL subtype classification remains based on cell of origin and genetic abnormatilies [2, 4]. These data highlight the need to routinely perform both IHC and FISH studies at the time of diagnosis and preferentially also at the time of recurrence [8].

### 2.1 DLBCL NOS Subtypes, Cell of Origin

A landmark study evaluated the gene expression profiling (GEP) of 96 normal and DLBCL lymphocytes and identified three unique genetic signatures with distinct patterns of somatic mutations [9]. Germinal center B cell‐like (GCB) DLBCL has a gene expression profile characteristic of normal germinal center B cells with intraclonal heterogeneity, ongoing somatic hypermutation, and CD10 and BCL6 expression. The activated B‐cell like (ABC) subtype has a gene expression profile of post‐germinal or activated B cells with high expression and constitutive activity of the nuclear factor kappa B (NF‐KB) complex and expression of IRF4 and BCL2. The third subtype is the unclassified subtype and accounts for 10%–15% of cases [8].

Gene expression profiling remains the gold standard to determine cell of origin (COO) but is not currently feasible in routine clinical care. The capacity to perform GEP routinely on fresh frozen samples is limited, and immunohistochemical (IHC) algorithms have been the most common method to determine COO in clinical practice. The Hans algorithm, which utilizes CD10, BCL6, and MUM1/IRF4, remains the most commonly utilized immunohistochemistry surrogate for GEP [10]. Each of these three IHC stains is considered positive if tumor cell expression is at least 30%. Unfortunately, concordance between the Hans algorithm and GEP is only 72%–86% [11, 12, 13, 14].

More recently, novel platforms such as the Lymph2Cx allow for digital GEP on fixed, paraffin‐embedded tissue. Though its use is restricted mostly to research, several studies have shown better concordance with GEP than IHC [8, 15, 16].

At this time, COO by gene expression profiling has often demonstrated poorer outcomes for the ABC‐DLBCL NOS [11, 12, 13, 14, 17, 18]. In a retrospective study of 157 de novo DLBCL cases treated with an up‐front rituximab chemo‐immunotherapy regimen, patients with the ABC subtype as identified by GEP had worse 5‐year PFS and OS compared to those with GCB subtype (31% vs. 76% and 45% vs. 80%, respectively) [15]. Another study of 344 patients with de novo DLBCL treated with R‐CHOP evaluated the impact of COO determined by Lymph2Cx assay on FFPE reported similar results with the 5‐year PFS and OS of the ABC subtype group being 48% and 56% versus 73% and 78% in GCB subtype [16]. Efforts to more accurately define COO are important, especially due to implications for response to therapy as well as the potential for increased risk of SCNSL in non‐GC COO DLBCL [19].

### 2.2 Molecular Features

C‐MYC is a proto‐oncogene located in chromosome 8q24. Ten to 15% of patients with newly diagnosed DLBCL have an underlying MYC rearrangement, resulting in dysregulated cellular survival and proliferation. Approximately half of these cases also carry a rearrangement of the anti‐apoptotic proto‐oncogene BCL2 and/or its transcription repressor BCL6. These genetic rearrangements are identified by fluorescent in situ hybridization (FISH). Their presence defined a DLBCL subset known as double‐hit or triple‐hit lymphoma, recognized in the 2014 WHO classification as High‐grade B‐cell lymphoma with MYC and BCL2 and/or BCL6 rearrangements (HGBCL‐DH/TH) [20]. These patients accounted for 8%–10% of de novo DLBCL diagnoses, have more aggressive disease and a worse prognosis after frontline treatment with R‐CHOP, especially in patients with advanced‐stage disease [21, 22]. However, even within this group, there is further heterogeneity [8].

Ennishi et al. performed a comprehensive analysis of RNA sequencing data from 157 patients with GCB DLBCL treated with up‐front R‐CHOP [23]. They established a Double‐Hit gene expression signature (DHITsig) able to identify a high‐risk subset of GCB cases (27%). This DHITsig group had a 5‐year time to progression rate of 57% compared with 81% for the rest of the cohort. HGBCL‐DH/TH with BCL2 rearrangements accounted for only 50% of the high‐risk DHITsig group. A subsequent study using whole‐genome sequencing showed the presence of cryptic rearrangements of MYC or BCL2 not detectable by routine testing within the DHITsig+ that may account for underlying MYC dysregulation in these patients [24].

Notably, GCB is enriched for DH/TH subtypes. It is important to note that the prognostic impact of MYC‐rearrangements depends on the translocation partner. The negative prognostic impact of *MYC*‐R is largely observed when *MYC* is translocated to an IG partner. *Lastly, MYC*/*BCL6*‐DH are rather heterogeneous in their molecular subtypes and show a mutation profile remarkably different from those with *MYC*/*BCL2‐*DH/TH with no prominent expression signatures, although a proportion of these cases are associated with *NOTCH2* mutation [25, 26]. The prognosis for MYC‐BCL6‐DH lymphoma is also likely better than for MYC‐BCL‐2 DH [27, 28, 29, 30, 31]. Therefore, the 5th edition of the WHO classification excludes the cases with concomitant *MYC* and *BCL6* rearrangements (without *BCL2* rearrangement) from the DH entity.

### 2.3 Protein Expression and COO

CD5+ DLBCL accounts for about 5%–10% of DLBCL‐NOS and is associated with a poorer prognosis [2, 32, 33]. It is more commonly noted in Asian patients.

Patients with DLBCL can also have a “double expressor lymphoma” (DEL), characterized by overexpression of the c‐MYC oncogene and BCL2 detected by IHC (≥ 40% and > 50%, respectively) [8]. DEL accounts for approximately a third of de novo cases and is also associated with a poorer prognosis [17, 34, 35, 36]. DELs can also be detected in up to 50% of relapsed refractory DLBCL, where they are also associated with poorer outcomes with salvage chemotherapy and autologous stem cell transplantation [37]. A report suggests DEL outcomes may result from the presence of underlying molecular changes, such as TP53 mutations and BCL6 translocations [38].

ABC‐DLBCL is enriched in both CD5+ DLBCL as well as DEL [2].

### 2.4 Genetic Subtypes

The use of whole‐exome sequencing further identified new genetic subtypes of disease characterized by frequently recurrent mutations [8].

Schmitz et al. analyzed 574 pre‐treatment DLBCL biopsy samples and identified four distinct genetic subtypes of disease with different recurring high‐frequency mutations [39]. These categories include the MCD, BN2, N1, and EZB subtypes. The MCD subtype was characterized by the co‐occurrence of MYD88(L265P) and CD79 mutations, the BN2 subtype by BCL62 fusions and NOTCH2 mutations, the N1 subtype had frequent NOTCH1 mutations, and the EZB subtype had EZH2 and BCL2 translocations. The MCD and N1 subtypes corresponded to ABC disease, whereas the BN2 and EZB subtypes corresponded to the GCB subtype. These groups portend different outcomes to upfront therapy. BN2 and EZB subtypes conferred a good prognosis, whereas the other subtypes conferred a poor prognosis.

In parallel, Chapuy and colleagues [40] classified 304 primary, previously untreated DLBCLs into five different DLBCL clusters. These include two distinct subsets of a low‐risk ABC‐DLBCL (C1 associated with MYD88 mutations), a poor prognosis ABC‐DLBCL (C5 that resembles the MCD subtype with MYD88‐L265P and CD79 mutations), an ABC/GCB‐independent group (C2 characterized by mutations and deletions of the chromosome 17p), a GCB‐DLBCL with poor and good risk (C3 and C4, respectively) [8]. Outside of the research setting, a probabilistic classification tool (LymphGen Genetic Subtype Classifier) has been developed that uses an algorithm to classify an individual patient's tumor based on the probability of belonging to a particular genetic subtype [41]. This tool is available online: https://llmpp.ccr.cancer.gov/lymphgen/userguide.php. The DLB*class* is another probabilistic molecular classifier for DLBCL that assigns a DLBCL to its respective C1‐C5 genetic subtype with 89% accuracy in the independent test set [42].

## 3 Risk Stratification and Prognostication

### 3.1 Clinical Features

Over the last three decades, the International Prognostic Index (IPI), which utilizes age > 60, stage, ECOG performance status > 1, and number of extranodal sites > 1, has been used to predict prognosis in aggressive NHL treated with doxorubicin‐containing regimens [43]. This score has been validated in the rituximab era (R‐IPI), where patients with a score of 0–1, 2, 3, and 4–5 had a 3‐year OS of 91%, 81%, 65% and 59%, respectively [44]. Finally, the NCCN‐IPI used age, LDH, sites of involvement, Ann Arbor stage and ECOG performance status to create four risk groups, with better low and high risk discrimination [8, 45]. A study comparing these three IPIs across 7 multicenter randomized trials utilizing R‐CHOP found that the NCCN‐IPI was best of the three at predicting OS. Unfortunately, none of these three scores identified a group with OS < 49% so all lack the ability to identify the highest risk DLBCL patients [46]. Despite this, the original IPI remains most commonly used for eligibility criteria to label “high risk” patients. The five‐year OS estimates in R‐CHOP treated patients ranged from 54%–88% using the IPI [46].

Another high‐risk subgroup within the IPI 1–2 cohort is bulky disease and/or very high LDH > 1.3xULN in patients 60 years or younger. This report validated findings in a US cohort for younger patients. Notably, these findings were not replicated for older patients [47, 48].

More recently, classifications based on COO and molecular features allow further identification of patient subsets with poor prognoses. In addition, several studies have reported the utility of PET imaging and circulating tumor DNA in the prognostication of patients with lymphoma.

### 3.2 Genetic Features

Section 2 reviews genetic features in particular, double‐hit lymphomas with MYC and BCL‐2 rearrangements that are associated with higher risk disease. Section 2 also reviews the impact of double expressor lymphomas.

Recent clinical trials have most commonly defined high risk LBCL as IPI 3–5 or double‐hit lymphoma.

### 3.3 PET/CT

PET/CT is the current preferred imaging modality for DLBCL staging and response assessment [49]. In clinical practice, the Deauville score, which is a visual assessment that compares lesional FDG uptake to the FDG uptake in liver, spleen, and mediastinal blood pool, is limited. This is especially the case for response assessment, particularly in interim imaging assessment, but also for end of treatment imaging. Although PET/CT has an excellent negative predictive value, its positive predictive value is lower due to its propensity for false positives [50] from infection, inflammation, trauma, and normal physiology. These limitations are demonstrated by the mixed results using the Deauville score visual assessment in determining early response to therapy [51].

Other measurements than the commonly used Deauville score may provide more accuracy. For example, the retrospective evaluation of the 360 patients from the phase 3 REMARC trial, which evaluated the addition of lenalidomide maintenance versus placebo in DLBCL patients age ≥ 60 years old treated with upfront R‐CHOP, used total metabolic tumor volume (TMTV) calculated as the sum of the metabolic volumes of all nodal and extranodal lesions [52]. A high TMVT, defined as > 220 at baseline PET, was able to identify patients with inferior EFS (HR 2.3, *p* = 0.0002) and OS (HR 3.3, *p* = 0.0001) when compared with those with lower TMVT. The prognostic ability of high TMVT was maintained across the different treatment groups, and after adjustment for LDH, B2‐microglobulin, performance status, and clinical risk scores (IPI and NCCN‐IPI) [8].

Another quantitative approach, the delta SUVmax, compares the SUV value of the most FDG‐avid lesions on baseline and interim scans and may improve reproducibility during response assessments. To this point, Schoder et al. recently reported the results of a prospective analysis of PET/CT serial evaluations of 504 patients studied in the phase 3 CALGB 50303 trial. They performed a comparison between visual Deauville 5‐point scale with percent change in FDG uptake (delta SUV) [53]. With a median follow‐up of 5 years, a delta SUV ≥ 66% on interim‐PET, measured after 2 cycles of chemotherapy, was predictive of OS (HR 0.21, *p* = 0.02) but not PFS. In contrast, visual assessment by Deauville score did not predict either outcome. The delta SUV value was also assessed in a phase 2 study of 1073 patients with newly diagnosed CD20+ lymphoma, including 609 with DLBCL [54]. Patients were treated with 2 cycles of R‐CHOP followed by an interim PET CT (iPET). A negative scan was defined as delta SUVmax > 66%. If the iPET was negative, patients were randomized to R‐CHOPx4 arm versus R‐CHOPx4 plus 2 cycles of rituximab arm. If the interim scans were positive, patients were randomized to an escalated Burkitt protocol arm or R‐CHOP × 6 arms. The iPET negative was negative in 87.5% of patients and positive in 12.5%. The post hoc analysis compared the deltaSUV method with the Deauville 5‐point scale. The study reported that iPET scan assessed by deltaSUV but not Deauville score accurately predicted better 2‐year PFS (79.4% vs. 36.7%, *p* < 0.0001) and 2‐year OS (88.2% vs. 59% *p* < 0.0001) in those patients with negative scans across all lymphoma types. However, escalation of cytotoxic therapy based on positive iPET did not translate into improved outcomes, similarly to several earlier trials, demonstrating the limitations of interim PET/CT in guiding therapy in DLBCL [8, 55].

### 3.4 Circulating Tumor DNA (ctDNA)

Circulating cell‐free DNA (cfDNA) is continuously released into the peripheral bloodstream by normal or tumor cells undergoing cell death. Measurable residual disease (MRD) strategies in DLBCL have used next‐generation sequencing (NGS) techniques to identify clonal tumor immunoglobulin heavy chain sequences (e.g., clonoSEQ; Adaptive Biotechnologies) or tracking of tumor‐specific mutations from a panel of disease‐specific genes—cancer personalized profiling by deep sequencing (CAPP‐Seq) [56]. Phased variant enrichment and detection sequencing (PhasED‐Seq) is a more sensitive assay because it identifies multiple mutations that occur together on the same strand of DNA, rather than assessing a single mutation [57]. This results in a lower background error rate, and so it allows for increased sensitivity of the test. Notably, unlike CLL, flow cytometry is not currently utilized for MRD due to the poor viability of LBCL lymphoma.

Advantages of monitoring cfDNA are its non‐invasive nature with the potential to track clonal evolution and detect new mutations that arise during treatment, which could be potentially exploited using targeted agents [8]. In a landmark study, Rochewski and colleagues retrospectively analyzed ctDNA in pre‐treatment tumor specimens, and serial serum samples of 126 patients with untreated DLBCL enrolled in three trials of upfront R‐EPOCH versus EPOCH [58]. CtDNA was analyzed using NGS by clonal VDJ rearrangements. After completion of treatment, patients were monitored with serial CT scans and concurrent serial serum samples. With a median of 11 years, positive ctDNA during surveillance had a positive predictive value of 88.2% and a negative predictive value of 97.8% for relapse. Patients developed detectable ctDNA with a lead time of 3.5 months prior to clinical progression. The ability of circulating tumor DNA (ctDNA) to detect early relapse has been confirmed since in several others studies, including in high‐risk patients [56], post‐allo‐HSCT [59], and in the RR setting in patients receiving CART‐therapy [60, 61], bispecific antibodies [62], and pola‐BR [63].

There are now prospective real‐world data validating the use of end‐of‐treatment (EOT) ctDNA MRD (PhasED‐Seq) status in frontline DLBCL treated with R‐CHOP or R‐EPOCH [64]. Patients with MRD‐positive EOT ctDNA had a 17% 3‐year PFS compared to 85% for patients who were MRD‐negative. MRD positivity had a higher PPV for 2‐year PFS than positive PET (68% vs. 56%) but a similar negative predictive value compared to PET/CT. Additionally, MRD positivity was associated with a higher risk of relapse within both subgroups with a complete metabolic response as well as a non‐complete metabolic response. These data support the role of MRD assessment in identifying high‐risk LBCL. These data support recent studies of prospective frontline clinical trials that demonstrate the utility of monitoring tumor‐specific phased variants over time to enhance prognosis over PET imaging [65].

Currently, clonoSEQ testing for patients with DLBCL is available for clinical use as a CLIA‐validated laboratory‐developed test, and it is covered by Medicare, although it is not FDA‐approved as it is in CLL. ctDNA continues to gain traction as an additional approach to DLBCL response assessment due to data suggesting it is more sensitive than standard‐of‐care imaging. In practice, the use of a ctDNA assay with the ability to detect less than 1 per one million may be considered to assist in interpretation if an EOT PET/CT is positive and if biopsy is not feasible [66].

The feasibility of using ctDNA for prospective decision‐making remains a challenge. Prospective trials may include either patients at high risk for relapse and thus who may benefit from consolidation, that is escalation of therapy, or potentially patients with excellent early outcomes, who might benefit from de‐escalation [57, 65]. Further study regarding ctDNA assay selection and frequency, type and interval of ctDNA assessment (single timepoint, rate of change, relation to treatment etc.) is needed.

### 3.5 Machine Learning

The use of machine learning to prognosticate treatment responses is a newly emerging field. There are new data demonstrating the feasibility of predicting responses to CAR‐T utilizing pretreatment baseline imaging using deep‐learning‐based image analysis [67]. This method was then validated using baseline PET/CT images from the JULIET trial, a phase 2 trial of tisagenlecleucel in patients with relapsed/refractory LBCL [68]. There are multiple potential uses of artificial intelligence including, in the imaging realm using advanced image reconstructions, improved ease of quantifying metrics such as total lesional glycolysis or total metabolic tumor volume, or predicting clinical outcomes based on imaging [69]. Implementation must address limitations including integration into clinical workflow, addressing AI‐induced errors, and the need for large‐scale validation and harmonization.

## 4 Frontline Therapy

DLBCL is an aggressive but curable disease for most patients, with survival rates similar to the general population in patients who have remained disease‐free for 2 years after frontline therapy (Table 1) [70, 71].

**TABLE 1:** Currently available therapies for advanced diffuse large B cell lymphoma and supporting data.

Regimen/phase	Population	PEP	F/U	OS	ORR	CRR	PFS	DOR	Notable toxicities
GELA LNH 98.5/III [72, 246] R‐CHOP versus CHOP	Untreated, 60–80 years, Stage II–IV	EFS	24 m 10 years	70% versus 57%	83% versus 69%	76% versus 63%	43% versus 61% 36.5% versus 20%	Relapses after 5 years were 7% of all PD	
POLARIX/III [73, 90] Pola‐R‐CHP versus R‐CHOP	Untreated, IPI 2–5	EFS	64.1 m	82.3% versus 79.5%	85.5% versus 83.8%	78% versus 74%	64.5% versus 59.1%	N/A	Fewer secondary malignancies in Pola‐R‐CHP (*n* = 5) versus (*n* = 12)
—/II* [247] DA‐EPOCH‐R	Untreated	NA	62 m	73%	100%	92%	70%	NR	G3 neutropenia 34%, G4 neutropenia 15%, febrile neutropenia 8%
ZUMA‐7/III [166, 167] Axicabtagene ciloleucel versus CIT + ASCT	Primary refractory or relapse < 12 m of 1 L	EFS	47.2 m	NR versus 31.1 m	83% versus 50%	65% versus 32%	14.7 m versus 3.7 m	NR	CRS 93%, G3+ 6%; ICANS 60%, G3+ 21%
ZUMA‐1/I/II [175, 248] Axicabtagene ciloleucel	R/R; 77% of patients refractory to 2 L+	ORR	63.1 m	25.8 m	83%	58%	5.9 m	11.1 m	CRS 93%, G3+ 11%; ICANS 64%, G3+ 30%
TRANSFORM/III [168, 169] Lisocabtagene maraleucel versus CIT + ASCT	Primary refractory or relapse < 12 m of 1 L	EFS	17.5 m	NR versus 29.9 m	87% versus 49%	74% versus 43%	NR versus 6.2 m (PFS) NR versus 2.4 m (EFS)	NR versus 9.3 m	CRS 49%, CRS G3+ 1%, ICANS 11%, ICANS G4 4%, no G4/5 CRS or ICANS
TRANSCEND/I/II [249, 250] Lisocabtagene maraleucel	R/R, after 2 L+	AE DLT ORR	19.9 m	27.3 m	73%	53%	6.8 m	23.1 m	CRS 42%, G3+ CRS 2%; ICANS 30%, G3+ ICANS 10%
JULIET/II** [171, 251, 252] Tisagenlecleucel	R/R, transplant ineligible or refractory after 2 L+	ORR	74.3 m	12 m	52%	40%	83% at 12 m	NR	CRS 58%, G3+ 22%, ICANS 21%, G3+ ICANS 15%
PARMA/III [205] High dose BEAC and ASCT versus DHAP	Relapsed intermediate or high‐grade chemosensitive DLBCL	EFS	63 m	53% versus 32%	84% versus 44%	NA	46% versus 12% (EFS)	NA	6% death rate in ASCT
NP30179/II [210, 212] Glofitamab	R/R after 2 L+	CR	12.6 m	50% at 12 m	52%	39%	37% at 12 m	18.4 m	CRS 63%, G2+ in 16%, ICANS 8%
STARGLO/III [181, 209, 213] Glofitamab‐GemOx versus R‐GemOx	R/R, transplant ineligible, after 1 L+	OS	20.7 m	25.5 m versus 12.9 m	68.3% versus 40.7%	58.5% versus 25.3%	13.8 m versus 3.6 m	NE versus 10.3 m	CRS 44%, mostly G1 (31.4%); ICANS 2.3%
EPCORE NHL1/II [181, 209, 213] Epcoritamab	R/R after 2 L+	ORR	49.2 m	18.5 m	59%	41%	4.2 m	20.8 m	CRS 51%, G3 3%
EPCORE NHL2/Ib [219] Epcoritamab‐GemOx	R/R CD20+ DLBCL after 1 L+	ORR	13.2 m	84.4% at 12 m	85.4%	61.2%	68.5% at 12 m	DOR CR 23.6 m	CRS 52.4%, G3+ 1%; ICANS 2.9%, G3+ 1%
L‐MIND/II [233, 234] Tafasitamab/Lenalidomide	R/R, after 1 L+	ORR	65.5 m	33.5 m	57.5%	41.3%	11.6 m	NR	Neutropenia G3+ 48%
SUNMO/III [188] Mosunetuzumab/polatuzumab versus GemOx	R/R, transplant ineligible, after 1 L+	ORR PFS	23.2 m	OS 50% versus 43% at 18 m	70% versus 40%	51% versus 24%	11.5 m versus 3.8 m	15.6 m versus 6 m	CRS 26%, G3+ 0.7%; infections 51%, G3+ 16%
GO29365/II [226]*** Pola‐BR versus BR	R/R, transplant ineligible, after 1 L+	CR	22.3 m	12.4 m versus 4.7 m	45% versus 17.5%	40% versus 17.5%	9.5 m versus 3.7 m	12.6 m versus 7.7 m	Higher rates of G3‐4 hematologic toxicity; neuropathy (43.6%) was G1‐2 and self‐limiting
POLARGO/III [228] Pola‐R‐GemOx versus R‐GemOx	R/R, transplant ineligible, after 1 L+	OS	N/A	19.5 m versus 12.5 m	52.7% versus 24.6%	40.3% versus 19.0%	7.4 m versus 2.7 m	N/A	G3‐4 AE rates similar (57.0% versus 58.4%), with higher rates of thrombocytopenia (34.4% vs. 26.4%), infections (14.1% vs. 8.0%) and PSN (57.0% vs. 28.8%) for Pola‐R‐GemOx
ECHELON‐3/III [232] BV ± len ± *R* versus Placebo + R^2^	R/R, transplant and CAR‐T ineligible, after 2 L+	OS	16.4 m	13.8 m (BVR ^2^) versus 8.5 m	64.3% (BVR ^2^) versus 41.5%	40.2% (BVR ^2^) versus 18.6%	4.2 m (BVR ^2^) versus 2.6 m	N/A	Neutropenia (46%) and diarrhea (31%)
LOTIS—2/II [230] Loncastuximab tesirine	R/R, after 2 L+	ORR	35 m	9.5 m	48.3%	24.8%	4.9 m	13.4 m	Grade 3+ neutropenia (26%), thrombocytopenia (18%), increased GGT (17%), and anemia (10%)
SADAL/II [239] Selinexor	R/R, transplant ineligible or refractory after 2 L+	ORR	11.1 m	9.1 m	28%	12%	2.6 m	9.3 m	G3‐4 hematologic toxicity in 92%

*Note:* All statistics are listed in order regimen is listed in the table. For example, R‐CHOP versus CHOP: ORR1 versus ORR2 corresponds to R‐CHOP ORR versus CHOP ORR. Toxicities listed are only for the superior arm when the trial is a randomized trial.

Abbreviations: 1L+, one or more lines of therapy; 2L+, two or more lines of therapy; AE, adverse event; ASCT, autologous stem cell transplant; B, bendamustine; BEAC, carmustine, etoposide, cytarabine, cyclophosphamide; BV, brentuximab vedotin; cyclophosphamide; CIT, chemoimmunotherapy; CRR, complete response rate; CRS, cytokine release syndrome; DLT, dose limiting toxicity; DOR, duration of response; EFS, event free survival; GemOx, gemcitabine‐oxaliplatin; GGT, gamma glutamyl transferase; ICANS, immune effector cell‐associated neurotoxicity syndrome; len, lenalidomide; m, months; N/A, not available; NE, not evaluable; NR, not reached; ORR, overall response rate; OS, overall survival; PEP, primary end point; PFS, progression free survival; pola, polatuzumab; R, rituximab; R/R, relapsed/refractory; R‐CHOP, rituximab, cyclophosphamide, doxorubicin, vincristine, prednisone; SAE, serious adverse event; SOC, standard of care; TEAE, treatment emergent adverse events; y, years.

* Negative phase III data comparing upfront RCHOP to DA‐EPOCH‐R in the Alliance/CALGB 50303 trial (Bartlett JCO 2019) precluded its FDA approval.

** Negative phase III data comparing tisagenlecleucel to SOC (CIT/ASCT) in the BELINDA trial (Bishop NEJM 2022) precluded its FDA approval in the second line.

*** Data reported are from the phase II randomized trial as assessed by independent review committee.

The standard frontline treatment of DLBCL is chemo‐immunotherapy with or without radiation according to disease stage and clinical risk factors. Patients with newly diagnosed DLBCL are generally classified as having either limited‐stage disease (Ann Arbor stage I or II without bulky disease or B symptoms) or advanced‐stage disease. The backbone of rituximab, cyclophosphamide, doxorubicin, and prednisone (R‐CHP) continues to remain the standard for most newly diagnosed DLBCL [72, 73].

### 4.1 Limited Stage

Early‐ or limited‐stage diffuse large B‐cell lymphoma (DLBCL) accounts for approximately half of all DLBCL cases [2, 3]. In the pre‐rituximab era, the SWOG S8736 trial established the use of a combined modality therapy using abbreviated chemotherapy (CHOP ×3) plus consolidative radiation therapy over CHOP ×8 as the standard of care for these patients [74]. However, long‐term follow‐up revealed a continued risk of relapse in both groups. With a median follow‐up time of more than 17 years, the PFS and OS of CHOP8 and CHOP3‐RT were similar (12 vs. 11.1 years, *p* = 0.73 and 13.0 vs. 13.7 years, *p* = 0.38) [75]. The addition of rituximab to CHOP3‐RT in SWOG S0014 improved outcomes with a 2‐year PFS of 92% and a 4‐year OS of 92% 0.27 Though the long‐term follow‐up data have not been published, the median PFS and OS were not reached at a median follow‐up time of 12 years [76].

Several trials have informed a positron emission tomography scan (PET) guided approach of abbreviated chemotherapy without radiation. In a LYSA/GOELAMS trial, 334 patients with stage I/II DLBCL, non‐bulky disease who achieved complete metabolic response (CMR) by PET after treatment with R‐CHOPx4 were randomized to receive consolidative radiation with 40Gy versus observation. The 5‐year survival was comparable in the radiation versus observation arms (PFS 92% vs. 89% and OS 92% vs. 96%) [77]. Underscoring that an abbreviated course of RCHOP alone without radiation may be sufficient for a select group of patients has been the FLYER study [78], a phase 3, multicenter non‐inferiority trial that enrolled 592 young patients (≤ 60 years) with stages I‐II, non‐bulky disease, normal LDH, and ECOG performance status of 0–1. The investigators compared R‐CHOP × 6 versus R‐CHOP × 4 followed by two doses of rituximab without radiation consolidation. After a median follow‐up of 5.5 years, the three‐year‐PFS for patients was 93% versus 96% for those treated with R‐CHOP × 6 versus R‐CHOP × 4 followed by two doses of rituximab, establishing 4 cycles of RCHOP as the standard of care for these low‐risk patients.

Using PET/CT after 3 cycles of RCHOP (iPET3), 158 patients with non‐bulky stage I/II DLBCL were enrolled and either received one further cycle of RCHOP if the iPET3 was negative or involved field radiation therapy followed by ibritumomab tiuxetan radioimmunotherapy. Eight‐9% of participants achieved a negative iPET3 and, with abbreviated therapy with R‐CHOP × 4 alone, achieved a 5‐year PFS of 87% and a 5‐year‐OS of 89% [79]. Unlike FLYER, S1001 included elderly patients (54% of study subjects were older than 60 years) and patients with adverse clinical characteristics (elevated LDH in 14% and smIPI score ≥ 1 in 73%) [8].

Although prospective data are lacking, patients with MYC rearrangements may be an exception to these excellent outcomes. A retrospective study of patients with limited‐stage DLBCL with MYC rearrangements showed a lower two‐year PFS and OS of 78% and 86%, respectively, in patients receiving R‐CHOP or intensified immunochemotherapy regimens with or without consolidative radiation per physician discretion without clear association of survival and therapy intensity [80]. Another retrospective registry study from the Netherlands identified 1434 limited stage patients with known *MYC*‐R status who were treated with R‐CHOP (−like) regimens. Fifty‐one % (*n* = 733) had stage I disease, and 49% (*n* = 701) Stage II. Stage I patients with (*n* = 83, 11%) and without (*n* = 650, 89%) a MYC‐R had similar 2‐year PFS (89% and 93%, *p* = 0.63) and OS (both 95%, *p* = 0.22), whereas for Stage II DLBCL patients with a MYC‐R (*n* = 90, 13%) inferior survival outcomes were seen when compared to Stage II patients without a MYC‐R (*n* = 611, 87%) (PFS 70% vs. 89%, *p* = 0.001; OS 79% vs. 94%, *p* < 0.0001). Furthermore, single MYC‐R and concurrent BCL2 and/or BCL6 rearrangements were associated with increased mortality and relapse risk. The authors concluded that there was no prognostic significance for MYC‐R in stage I disease, whereas in stage II disease, MYC‐R was negatively associated with survival [81].

Nevertheless, current data support the option for an abbreviated course of chemo‐immunotherapy for limited‐stage DLBCL in the majority of patients [8, 82, 83].

### 4.2 Advanced Stage

**FIGURE 1:**
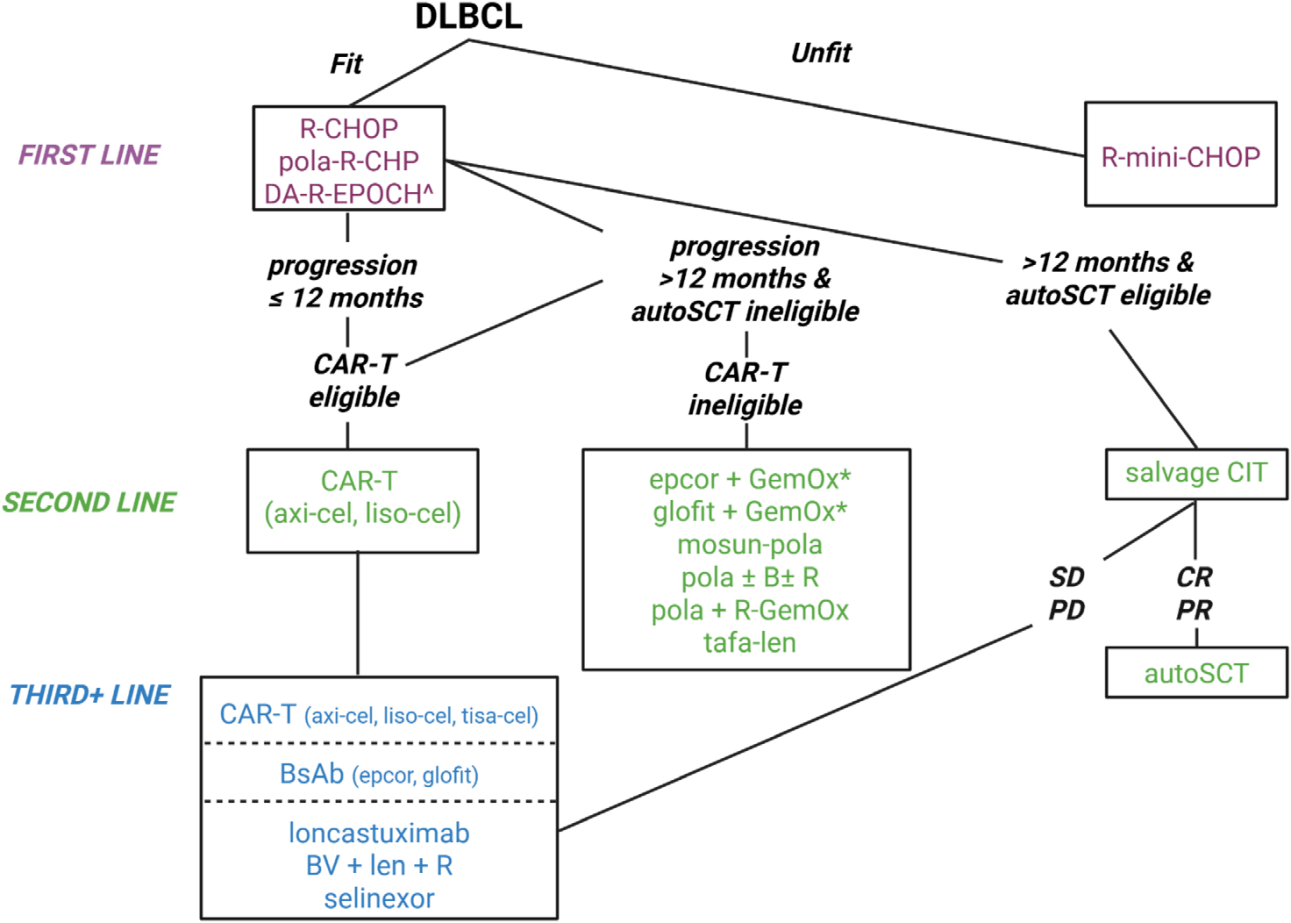
Our approach to therapy of DLBCL. Third line therapies in order of preference are: CAR‐T if not already received, then bispecific antibody combination therapy as outlined in CAR‐T ineligible second line options or bispecific antibody monotherapy, followed by the remainder of listed therapies in both the second‐line CAR‐T ineligible group as well as the third‐line therapy group. All treatments are listed in alphabetical order unless otherwise indicated in the figure or legend. Other options for third‐line DLBCL in someone who is CAR‐T and/or transplant ineligible, or experienced relapse after CAR‐T include ibrutinib and lenalidomide ± rituximab for non‐GC DLBCL. Abbreviations: *, on NCCN guidelines v1.2026, but not FDA‐approved as of January 1, 2026; ^, for DLBCL or HGBCL with MYC and BCL2 gene rearrangements; axi‐cel, axicabtagene ciloleucel; B, bendamustine; BsAb, bispecific antibody; BV + len + *R*, brentuximab vedotin, lenalidomide, rituximab; CAR‐T, CD19‐directed chimeric antigen receptor‐modified T‐cells; CIT, chemoimmunotherapy; CR, complete response; DA‐R‐EPOCH, dose‐adjusted‐R‐EPOCH; DLBCL, diffuse large B‐cell lymphoma; epcor, epcoritamab; GemOx, gemcitabine, oxaliplatin; glofit, glofitamab; liso‐cel, lisocabtagene maraleucel; mosun, mosunetuzumab; PD, progressive disease; pola, polatuzumab; PR, partial response; R, rituximab; R‐CHOP, rituximab, cyclophosphamide, doxorubicin, vincristine, prednisone; R‐CHP, rituximab, cyclophosphamide, doxorubicin, prednisone; SD, stable disease; tafa‐len, tafasitamab‐lenalidomide; tisa‐cel, tisagenlecleucel. Figure created with Biorender.

Advanced stage DLBCL accounts for about half of patients with DLBCL [2, 3]. After two decades without improvement in R‐CHOP outcomes [84, 85, 86, 87, 88, 89], polatuzumab (pola) in combination with R‐CHP has joined R‐CHOP as a standard of care for first‐line, curative intent therapy. There is no clear consensus for optimal management of frail patients (Figure 1, Table 1).

#### 4.2.1 Fit Patients, Frontline Standard of Care

Most fit patients receive frontline therapy with either pola‐R‐CHP or R‐CHOP (rituximab, cyclophosphamide, doxorubicin, prednisone) [72, 73].

The phase III POLARIX study (Table 1) randomized patients with previously untreated DLBCL, NOS, or high‐grade B‐cell lymphoma (HGBL) with an IPI of 2–5 and an ECOG of 0–2 to either R‐CHOP versus polatuzumab‐R‐CHP. Polatuzumab is an antibody‐drug conjugate (ADC) comprised of a CD79b‐directed antibody linked to monomethyl auristatin E (MMAE), which is cytotoxic. The rationale for substituting polatuzumab for vincristine in pola‐R‐CHP was due to potential overlapping peripheral sensory nervous system toxicity. In April 2023, pola‐R‐CHP received FDA approval for untreated DLBCL NOS or HGBCL with an IPI of 2 or greater. This FDA approval was based on improved PFS compared to R‐CHOP at 2 years [73], and this is now confirmed at 5 years follow‐up; 5‐year updated PFS rates were significantly higher with pola‐RCHP at 65% versus 59%. With the five‐year update, there was still no statistically significant difference in overall survival [90]. Exploratory subgroup analyses showed continued benefit from pola‐R‐CHP in the same groups that appeared to most benefit in the 2 year follow‐up: IPI score 3–5, multiple extranodal sites of disease, ABC‐DLBCL by gene expression profiling, and double expressor lymphoma by immunophenotyping. Practically, utilizing GEP to determine COO is not feasible in routine practice and use of IHC as a surrogate misclassifies in 20%–30% of DLBCLs [10, 91] In support of the unreliability of IHC to determine COO, real world studies of frontline pola‐RCHP utilized IHC found similar OS and CRR in GC versus non‐GC DLBCL [92, 93]. Although there were numerically fewer deaths from progressive lymphoma in the pola‐RCHP arm, this was not statistically significant [90].

Outside of the ABC subtype, it remains unclear whether other biomarker defined DLBCL subtypes, including germinal center B‐cell with a dark zone signature and HGBCL, also benefit similarly from Pola‐RCHP [94], but the POLARIX results establish Pola‐RCHP as a SOC for the upfront treatment for advanced DLBCL.

##### 4.2.1.1 Double Hit/Triple Hit Lymphoma

The optimal treatment of double hit lymphoma (DLH) and triple hit lymphoma TLH remains undefined. A retrospective series of 129 patients with DLH demonstrated inferior event free survival with RCHOP (25%) and R‐HyperCVAD/MA (rituximab, hyperfractionated cyclophosphamide, vincristine, doxorubicin, dexamethasone, alternating with cytarabine plus methotrexate) (32%) compared with those who received DA‐EPOCH‐R (dose‐adjusted etoposide, prednisone, vincristine, cyclophosphamide, doxorubicin plus rituximab) (67%) [95]. Despite a paucity of randomized prospective trials in DHL and THL, treatment with DA‐EPOCH‐R is often the preferred regimen for fit patients with advanced disease with consideration of ISRT for localized disease. Other treatment options for DHL and THL with potential for increased toxicity include R‐HyperCVAD and CODOX‐M/IVAC‐R (cyclosphosphamide, vincristine, doxorubicin, methotrexate alternating with iphosphamide, etoposide, cytarabine, and rituximab) [96]. A retrospective study in patients aged 60 or under with DHL or THL compared CODOX‐M/IVAC‐R versus DA‐EPOCH‐R and demonstrated higher complete response rates in the CODOX‐M/IVAC‐R group (80%) compared with DA‐EPOCH‐R (58%). Although the CODOX‐M/IVAC‐R group also had prolonged event free survival at 1, 2, and 5 years, there was no difference in overall survival between the two groups [97]. The efficacy of polatuzumab in DHL and THL is uncertain; while these patients were included in POLARIX, there were only 19 (5.7%) patients with DHL/THL in the RCHOP arm and 26 (7.9%) patients with DHL/THL in the R‐Pola‐CHP arm, limiting the evaluation of R‐Pola‐CHP in these higher risk populations [73].

##### 4.2.1.2 Double Expressor Lymphoma

Patients with double expressor lymphoma (DEL) often have ABC subtype of DLBCL and typically present over age 60 with advanced stage and intermediate‐to‐high IPI [98]. As such, many patients with DEL are treated with R‐Pola‐CHP which has an ORR in the DEL population of 83.3% and a CRR of 72.2% with hematologic toxicities as the main adverse event with 45.3% of patients developing neutropenia after 3 cycles [99]. Retrospective studies of RCHOP versus DA‐EPOCH‐R in DEL yield conflicting results [100], however the largest analysis of 155 DEL patients demonstrated no difference in 3‐year PFS (57.2% vs. 33.2%, *p* = 0.063) or OS (71.6% vs. 72.2%, *p* = 0.43) [101]. Among patients with lower risk DEL (IPI < 2), R‐CHOP may be considered.

#### 4.2.2 Fit Patients, New Frontline Approaches (COO Agnostic)

Numerous clinical trials are continuing the tradition of adding agents to the standard chemoimmunotherapy backbone for high‐risk diffuse large B cell (Table 2). High‐risk LBCL has poor outcomes, with CRR as low as 40%–60% [25, 36, 44, 95, 96, 102, 103]. Bispecific antibody‐chemotherapy combinations, immunomodulatory agents, and CAR‐T trials are under investigation in frontline DLBCL.

**TABLE 2:** Selected clinical trials for diffuse large B cell lymphoma.

Concept	Phase/setting	NCT #	Design
Reducing frontline chemotherapy in fit patients	II/1 L	NCT03960840	2 cycles of 1 L therapy then rapcabtagene autoleucel (YTB323)
	III/1 L ZUMA‐23	NCT05605899	R‐CHOP/DA‐R‐EPOCH versus 2 cycles of chemotherapy + axicabtagene ciloleucel.
Frontline R‐CHOP + X	III/1 L GOLSEEK‐1	NCT06356129	Golcadomide + *R*‐CHOP versus placebo + *R*‐CHOP
	III/1 L FrontMIND	NCT04824092	R‐CHOP+tafa/len versus R‐CHOP
	III/1 L OLYMPIA‐3	NCT06091865	Odronextamab + CHOP versus R‐CHOP
	III/1 L EPCORE DLBCL‐2	NCT103378976	Epcoritamab + *R*‐CHOP versus R‐CHOP
	III/1 L SKYGLO	NCT06047080	Glofitamab‐pola‐R‐CHP versus pola‐R‐CHP
Frontline R‐mini‐CHOP + X	III/1 L SWOG1918	NCT04799275	Azacitadine + mini‐R‐CHOP versus mini‐RCHOP. Stratification by EPI
	III/1 L POLAR BEAR 80+ or 75 with frailty by CGA	NCT04332822	Mini‐R‐CHOP versus pola‐R‐mini‐CHP × 6
	III/1 L ARCHED Elderly > 80 years or 61–80 years and unfit for R‐CHOP	NCT05820841	Randomized trial of acalabrutinib + *R*‐mini‐CHOP versus R‐mini‐CHOP. Stratigication by age, ADL score, IPI
	II/1 L elderly ineligible for R‐CHOP	NCT04663347	Epcoritamab‐R‐mini‐RCHOP × 6 cycles +2 additional cycles of epcoritamab monotherapy
Omitting chemotherapy frontline in elderly/unfit/frail	III/1 L Elderly > 70 years, unfit/frail	NCT05179733	ZR2 versus R‐mini‐CHOP
	II/IL Ineligible for R‐CHOP	NCT05798156	R‐pola‐glofit × 6 cycles, then 6 cycles consolidation
	II/1 L MorningSun; ≥ 80 years or 65–79 and ineligible for R‐CHOP	NCT05207670	Subcutaneous fixed duration mosunetuzumab for up to 17 cycles (1 year)
	II/1 L EPCOR DLBCL‐3 ≥ 80 years or≥ 75 years with comorbidities	NCT05660967	Subcutaneous fixed duration epcoritamab for up to 12 cycles (1 year)
	II/1 L ACRUE ≥ 80 years or 61–79 and chemoimmunotherapy inegligible	NCT05952024	Rituximab for 8 cycles and acalabrutinib for 28 cycles
Bispecific antibodies after CAR‐T	Post CART	NCT04703686	≥ 1 months, first SD/PD; glofitamab × 11 months
	Post CART	NCT04889716	Day 30 PR/SD/PD: mosunetuzumab or glofitamab
	Post‐CART	NCT06238648	Randomized if Day 30 PR: Epcoritamab versus observation
	Post CART	NCT05633615	Randomized if Day 30 PR/SD: mosunetuzumab or polatuzumab or mosunetuzumab/polatuzumab or observation
	Post CART	NCT06552572	Month 1 or Month 3 PR; glofitamab × 12 months
Bispecific antibodies before and after CAR‐T	Pre/Post CART	NCT06071871	Pre: Glofitamab‐polatuzumab‐obinutuzumab bridging Post: If no CR or PD post CART
	Pre/Post CART	NCT05260957	Pre: Mosunetuzumab/polatuzumab bridging Post: Mosun
	Pre/Post CART	NCT06213311	Pre: Glofitamab bridging Post: Day 30 glofit
	Pre/Post CART	NCT06458439	Pre: Epcor bridging Post: Day 30 epcor
	Pre/Post CART	NCT06854159	Pre: Odronextamab bridging Post: Odronextamab
Bispecific antibodies for bridging pre‐CAR‐T	Pre CART	NCT06784726	Pre: Odronextamab bridging
	Pre CART	NCT06834373	Pre: golcadomide + *R* bridging
	Pre CART	NCT04512716	Pre: 131‐I‐Apamistamab (RIT)
	Pre CART	NCT05800405	Pre: Radiation therapy bridging
Bispecific antibodies + ADC	RR	NCT05672251	Mosunetuzumab + loncastuximab
	RR	NCT06015880	Mosunetuzumab + polatuzumab + Lenalidomide
	RR	NCT03533283	Glofitamab + polatuzumab
	RR	NCT04970901	Loncastuximab + glofitamab, etc.
Bispecific antibodies + CIT	RR	NCT05364424	Glofitamab + *R*‐ICE
	RR	NCT04663347	Epcoritamab + DHAX/C, autoSCT eligible
CAR‐T versus ASCT after bispecific antibodies	RR	NCT05852717	Epcoritamab + GDP then CART or auto SCT
ADC + CIT	RR	NCT04384484	Loncastuximab‐R versus CIT
	RR	NCT05139017	Zilovertamab vedotin + *R*‐Gem/Ox
	RR	NCT04182204	Polatuzumab + *R*‐Gem/Ox versus R‐Gem/Ox
	RR	NCT04833114	Polatuzumab + *R*‐ICE versus R‐ICE
Novel bispecific antibodies	RR	NCT04594642	AZD0486 3L+ (CD19 × CD3)
	RR	NCT06526793	AZD0486 (SOUNDTRACK‐B) (CD19 × CD3)
Novel CAR‐T	RR	NCT04792489	Zamtocabtagene Autoleucel (CD19, CD20, fresh), Phase 2
	RR	NCT03287817	AUTO3 dual CAR: CD19, CD22
	RR	NCT04182204	bbT‐369 dual CAR: CD79a, CD20
	RR	NCT06208735	CLIC‐2201 (CD22 CAR‐T)
	RR		CD22 (CARGO)
	RR	NCT04637763	CB‐010 (allogeneic anti‐CD19 CAR T TRAC KO, PD1 KO)
	RR	NCT05370430	BAFFR CAR‐T
Enhancing CAR‐T or bispecifics	RR	NCT06271057	Golcadomide + CAR‐T
	RR	NCT06508658	Epcoritamab + lenalidomide versus R‐Gem/Ox
	RR	NCT06508658	Englumafusp alfa (CD19‐4‐1BBL) + glofitamab
Targeted therapy	RR	NCT02077166	Ibrutinib + lenlidomide + rituximab
	RR	NCT04572763	Copanlisib + venetoclax
	RR	NCT05618366	Tazemetostat + venetoclax

Abbreviations: 1L, 1st line therapy; autoSCT, autologous stem cell transplant; CR, complete response; DA‐R‐EPOCH, dose‐adjusted rituximab, etoposide, prednisone, vincristine, cyclophosphamide, doxorubicin; epcor, epcoritamab; emOx, gemcitabine‐oxaliplatin; ICE, ifosphamide, carboplatin, etoposide; mosun, mosunetuzumab; PD, progressive disease; pola, Polatuzumab; PR, partial response; R, rituximab; R‐CHOP, rituximab, cyclophosphamide, doxorubicin, vincristine, prednisone; SD, stable disease; tafa/len, tafasitamab and lenalidomide; ZR2, zanubrutinib, lenalidomide, rituximab.

Multiple trials are underway combining bispecific antibodies with an R‐CHP backbone. The COALITION study [104] enrolled younger patients (≤ 65 year olds) with LBCL and at least one high risk feature (IPI ≥ 3, NCCN‐IPI ≥ 4, or rearrangements of MYC and BCL2 and/or BCL6). This population has been characterized as having an expected 46%–58% 5‐year PFS with R‐CHOP [46] (CITE Ruppert Blood 2020). Eighty patients received 1 cycle of R‐CHOP to allow enrollment of patients who urgently required therapy. Patients were then randomized 1:1 to 5 cycles of glofit‐pola‐R‐CHP or Glofit‐R‐CHOP, followed by two additional cycles of glofit consolidation. Cytokine release syndrome (CRS) occurence was low (21% of patients) and all CRS was grades 1–2. The CRR was 98%, 2‐year PFS was 86%, and 2‐year OS was 92%. Notably, patients who had a PR at the end of induction all ultimately achieved a complete metabolic response and ctDNA clearance continued to occur after end of induction during glofitamab consolidation [104]. Given this promising safety and efficacy, the SKYGLO trial (NCT: 06047080) will be important to elucidate the contribution of glofitamab to upfront chemoimmunotherapy; this trial compares glofitamab‐pola‐R‐CHP to pola‐R‐CHP in patients of all ages with IPI 2–5 [105].

Unlike these results, a 62‐patient trial of mosunetuzumab+pola‐R‐CHP versus pola‐R‐CHP did not show clear benefit with the addition of mosunetuzumab in first‐line DLBCL; CR rates were comparable (73% versus 77%) and the 2‐year PFS did not demonstrate clinical benefit M‐pola‐R‐CHP 82% (95% CI: 66%–98%) versus pola‐R‐CHP 71% (95% CI: 56%–86%) (NCT03677141) [106].

Three year outcomes were also reported for the frontline epcoritamab‐R‐CHOP phase II trial that enrolled high risk patients, defined as IPI 3–5, DHL/THL, or bulky disease > 10 cm [107]. Epcoritamab was administered weekly for the first 4 cycles, then every 3 weeks for cycles 5–6 in combination with SOC R‐CHOP, and then epcoritamab monotherapy every 4 weeks for 1 year in total. Median time from diagnosis to first dose was 4 weeks [89] and 96% completed 6 cycles of R‐CHOP [108]. Forty‐six patients were evaluable and CRR was 85% with a 98% ORR [107]. At 33 months, 80% of patients remained progression‐free and 87% of patients were still alive; DOR was estimated at 67%; DOCR was 75% [107]. Two patients died from infection; other common AEs included CRS (60%; 45% gd 1, 11% gd 2, 4% gd 3), neutropenia (70%), and anemia (68%), fatigue (49%), nausea (47%), fever (43%), and injection‐site reaction (40%) [108]. Responses appeared comparable across IPI 3 versus 4–5, COO, and bulky versus non‐bulky disease. 91% of patients achieved uMRD (AVENIO ctDNA) [108]. Of note, only 68% of patients completed planned treatment; 11% discontinued therapy due to AEs (11%), 6% withdrew [107]. These data are the basis for the EPCORE DLBCL‐2 phase 3 trial comparing epcoritamab + R‐CHOP versus R‐CHOP (NCT103378976) for frontline DLBCL.

Finally, the OLYMPIA‐3 (NCT06091865) trial, a Phase 3 randomized trial comparing odronextamab + CHOP to R‐CHOP in untreated DLBCL, reported results of their dose escalation cohort of odronextamab + CHOP [109]. Patients were required to have LBCL with IPI ≥ 2 or DHL/THL and 22 patients were enrolled. 68% of patients completed planned treatment, a number strikingly similar to the experience with epcoritamab‐R‐CHOP. Impressively, ORR and CRR in the dose level 2 cohort (*n* = 13) were 100%. Survival follow‐up was immature at 6 months. Importantly, this is the only frontline bispecific antibody‐chemotherapy study that does not include monoclonal antibodies. Thus, these data provide support for the potential to omit monoclonal anti‐CD20 antibodies when chemotherapy is administered in combination with bispecific antibodies. Longer follow‐up and the dose optimization results will be critical.

The incorporation of immunomodulatory agents into frontline DLBCL treatment represents an additional therapeutic strategy to improve outcomes, although data are inconsistent. The addition of lenalidomide to R‐CHOP improved PFS and OS in the randomized Phase II ECOG‐ACRIN E1412 trial [110], but not in the Phase III ROBUST trial [111]. Both trials used NanoString Lymph2Cx to determine COO; however, notable differences included a lower number of high‐risk patients, different lenalidomide dosing (len 25 D1‐10 ECOG vs. len 15 mg D1‐14 ROBUST), and longer time from diagnosis to treatment in ROBUST.

The First‐MIND Phase 1b study studied tafasitamab and tafasitamab/lenalidomide combinations agnostic of COO; both approaches were safe, and efficacy appeared better in the tafa/len arm [112]. FrontMIND (NCT04824092) is currently enrolling; this Phase 3 randomized double‐blind placebo compares R‐CHOP+tafa/len to R‐CHOP in untreated high‐intermediate and high‐risk DLBCL. The primary endpoint is PFS [113].

Golcadomide, an oral, small molecule agent that is a cereblon E3 ligase modulator (CELMoD), is another promising new immunomodulatory drug. It binds to cereblon, a substrate recognition adapter in the CRL4^CRBN^ E3 ubiquitin ligase complex cellular machinery that tags proteins for destruction by a proteosome [114, 115]. Binding to cereblon induces the closed, active conformation of cereblon to recruit, ubiquitinate, and induce degradation of Ikaros and Aiolos, which are critical for survival of B‐cell malignancies, triggering direct tumor killing and immunomodulatory activity [116]. Notably, the efficacy of golcadomide is COO agnostic.

CC‐220‐DLBCL‐001 (NCT04884035) is a Phase 1b multicenter trial examining safety of Golca + R‐CHOP for untreated aggressive B‐cell lymphoma. Two‐year efficacy outcomes were reported [116]. Thirty‐three patients were assessed at the recommended phase 2 dose of 0.4 mg D1‐7. CMR was 88% (29/33) and all patients remained progression free at 24‐month efficacy assessment cutoff. High risk patients were defined as IPI 1–2 with HR features (≥ 1 bulky lesion 7 cm and/or LDH > 1.3 ULN) or IPI 3–5. The complete metabolic response rate was 89% with similar CRR in GC (94%; 16/17) and non‐GC (82%, 9/11) patients with high risk DLBCL. Notably, grade 3–4 AEs were primarily hematologic; 87% had neutropenia (23% febrile neutropenia), 42% with thrombocytopenia, and 36% with anemia. Non‐hematologic adverse events including fatigue, rash, GI toxicity were low grade and uncommon [116]. This approach will be further studied in the GOLSEEK‐1 (NCT06356129) phase 3, double‐blind trial for frontline golcadomide in high risk LBCL. Patients will be randomized 1:1 to Golca + R‐CHOP × 6 cycles or placebo + R‐CHOP × 6 cycles. The primary endpoint is investigator assessed PFS [117].

Clinical trials aiming to remove or reduce chemotherapy in the frontline DLBCL setting support this proof‐of‐concept and also hint at the possibility that the field may be able to move beyond chemotherapy for untreated DLBCL in the not‐too‐distant future.

The Smart Stop trial (NCT04978584) [118] assessed the ability to reduce or remove chemotherapy following an initial response to targeted therapy. Patients with untreated LBCL received lenalidomide, tafasitamab, rituximab, and acalabrutinib (LTRA) for four 21‐day cycles, followed by PET/CT response‐adapted therapy. All patients then received an additional 6 cycles of LTRA with or without CHOP based on response and randomization. If patients did not achieve CR, then they received LTRA+CHOP × 6 cycles (groups B and D). For patients who achieved a CR after 4 cycles of LTRA, cohort 1 was given 2 cycles of CHOP plus LTRA × 6 (group A); in Cohort 2, patients in CR continued without CHOP for LTRA × 6 (group C). Median follow‐up was 22 months. Sixty‐two patients were enrolled. 56% of patients had poor risk IPI. After 4 cycles of LTRA, ORR was 90% with 57% CRR. Twenty‐four month estimated PFS was 85% for the entire group. For cohort A, of the 19 patients with CR after LTRA × 4, followed by CHOP × 2, one had progressive (non‐LBCL) and the 30‐month PFS is 100%. Of the 16 patients with CR after LTRA × 4 who did not receive any chemotherapy (cohort C), four patients had PD at a median of 21 months and were treated off‐protocol with R‐CHOP‐like therapy, all achieving CR. 18‐month PFS estimate was 92%. Outcomes for groups B and D have not yet been reported. Thus, in selected patients with LBCL in remission after targeted therapy, it is feasible to omit or reduce chemotherapy. Longer follow‐up will be reassuring to confirm that those patients who did not receive chemotherapy are cured.

Consolidation with CAR‐T also appears a feasible approach in newly diagnosed LBCL. The ZUMA‐12 trial was a phase 2 trial of axicabtagene ciloleucel for patients with high‐risk lymphoma (DHL, THL and IPI ≥ 3) and a positive interim PET/CT (Deauville 4 or 5) after 2 cycles of chemotherapy. Half of patients received R‐CHOP and the other half predominantly R‐EPOCH. ORR was 92% with 86% CR [119, 120]. Forty‐seven month follow‐up reported an estimated 75% PFS, 82% DOR, and 81% OS. No relapses were observed after 18 months from CAR‐T infusion. These results are the basis for the ZUMA‐23 trial currently underway [121]. This trial enrolls high risk LBCL and will compare 6 cycles of R‐CHOP/DA‐R‐EPOCH to 2 cycles of chemotherapy + axicabtagene ciloleucel. The primary endpoint is EFS.

Rapcabtagene autoleucel (YTB323) is also under investigation in a phase 2 trial for first‐line high‐risk LBCL [122]. This CAR‐T is a CD19‐directed CAR‐T that utilizes a short manufacturing time (< 2 days) to preserve Tcell stemness and improve in vivo expansion [123].

#### 4.2.3 Frontline Therapies for Fit Patients Targeting DLBCL Subgroups

As discussed, ABC DLBCLs are characterized by activation of the NF‐κB pathway and chronic B‐cell receptor signaling. Efforts to improve up‐front therapy in non‐GCB DLBCL have combined R‐CHOP with different biologic agents targeting NF‐κB pathway activation and BCR signaling, including ibrutinib, bortezomib, and lenalidomide, but these approaches have not translated into improved patient outcomes. Randomized, double‐blind placebo‐controlled phase III trials evaluating the combination of R‐CHOP plus ibrutinib for stage I–IV non‐GCB/ABC DLBCL (PHOENIX trial) [88] and R‐CHOP plus lenalidomide (R2CHOP) in non‐GCB/ABC IPI 2–5 DLBCL (ROBUST trial) [111] failed to demonstrate a significant improvement in outcomes over R‐CHOP alone. These results likely reflect heterogeneity within the COO subgroup. Additional subgroup analyses of these studies suggested that younger patients and/or those with overexpression of BCL‐2 and MYC (double expressor lymphomas) may have better outcomes with the addition of ibrutinib, but potential improvements in lymphoma‐specific outcomes may have been negated by increased toxicities and less R‐CHOP dose intensity in older patients.

Studies of second‐generation non‐covalent BTKi in combination with standard‐of‐care chemoimmunotherapy for DLBCL are underway (Table 2). Perhaps a less‐toxic second generation BTKi will overcome the toxicity observed when ibrutinib was combined with R‐CHOP and improve outcomes. The REMoDL‐A trial [124] is a randomized phase 2 trial of acalabrutinib+R‐CHOP versus R‐CHOP; they observed similar relative dose intensity between the two arms, albeit with a trend towards increased severe gastrointestinal toxicity in older patients over 65 years old; efficacy was not reported. The ESCALADE trial (NCT04529772) [125] builds upon experience with ibrutinib and R‐CHOP; this randomized phase 3 study of R‐CHOP + acalabrutinib versus R‐CHOP + placebo focuses on younger patients ≤ 65 years old with untreated non‐GC like DLBCL. Patients receive 1 cycle of R‐CHOP, then undergo gene expression profiling. If the tumor is non‐GCB DLBCL (i.e., ABC‐like or unclassified), patients are randomized from cycle 2 onwards to BTKi + R‐CHOP versus placebo + R‐CHOP. The primary endpoint is PFS.

Strategies for double expressor lymphoma (DEL) under study include the addition of histone deacetylase inhibitors (HDACi) and BTKi to R‐CHOP. The DEB study (NCT04231448) [126], undertaken in China, was a randomized phase 3 trial of tucidinostat, formerly chidamide, plus R‐CHOP compared to R‐CHOP alone in untreated DEL DLBCL. Patients who achieved a complete metabolic response received either tucidinostat for 24 weeks or placebo. Median follow‐up was over 3 years, with improved EFS at 3 years (57% vs. 48%). Orelabrutinib, a more selective BTKi, is also being studied in combination with R‐CHOP for DEL, although these data are immature [127].

Finally, new trials are attempting to implement genetic subgrouping of DLBCL to optimize frontline therapy. GUIDANCE‐01 trial [128] was a randomized Phase II trial of 128 patients who received R‐CHOP versus R‐CHOP plus one of five therapies selected based on genetic subtyping of each patient's newly diagnosed intermediate to high risk DLBCL. A 20‐gene algorithm similar to LymphGen categorized patients into 6 genetic subtypes (MCD‐like, BN2‐like, N1‐like, EZB‐like, TP53 mutated, and NOS). MCD‐ and BN2‐like groups were characterized by alterations in BCR and NK‐kappaB signaling, so this group received ibrutinib‐R‐CHOP. The EZB subtype, which often had mutations in epigenetic modifiers, received tucidinostat‐R‐CHOP. Decitabine was utilized for the TP53 mutated DLBCL, and the N1 and NOS groups received lenalidomide‐R‐CHOP. The matching strategy markedly improved efficacy outcomes, including CRR, and two‐year PFS and OS. These results are striking; however, major criticisms include that the trial utilized an unvalidated gene algorithm and was underpowered to demonstrate benefit for individual treatment subgroups [129]. Moreover, it was unblinded and conducted at a single Chinese center. The GUIDANCE‐02 randomized phase 3 trial aims to address this criticism with a much larger, 1100‐person trial utilizing a validated 38‐gene algorithm “LymphPlex.” [130] In the R‐CHOP‐X arm, patients with MCD‐like, BN2‐like, and N1‐like subtypes receive oral orelabrutinib (BTKi), patients with EZB‐like, ST2‐like, and NOS (not otherwise) receive oral lenalidomide, and patients with TP53 mut (TP53 mutations) subtype receive decitabine + *R*‐CHOP. The primary endpoint is PFS and the trial is currently ongoing with 58 sites in China. As of March 2025, 1043 of 1100 planned patients have been randomized [131].

The US National Clinical trials Network is planning a Phase 3 clinical trial in which patients with newly diagnosed DLBCL utilizing DLB*class* [132]. Patients will receive 1 cycle of standard of care therapy (pola‐R‐CHP or R‐CHOP) and then undergo genetic subtype categorization by DLBclass, then be randomized to frontline pola‐R‐CHP with or without a targeted agent.

#### 4.2.4 Frail Patients

##### 4.2.4.1 Standard of Care Approaches

The relative incidence of ABC DLBCL increases with age, likely due to underlying immunosenescence, which is a known phenomenon of aging [133, 134]. Decreased B cell receptor diversity is also observed in older individuals, and clonal expansion of B cells has been observed in vivo; thus, it is possible the malignant DLBCL clones grow in the background of clonal expansion of B cells associated with aging [135, 136]. EBV+ DLBCL is also common in the elderly population and is also mostly ABC DLBCL.

Clinical management of frail and/or elderly patients with comorbidities remains challenging. R‐CHOP or pola‐R‐CHP are the optimal regimens if patient fitness permits. Three‐year failure free survival in patients 60 years and older was 53% [137]. R‐mini‐CHOP is often utilized in patients who are frail or elderly with curative intent [138, 139, 140]. Outcomes with R‐mini‐CHOP appear inferior to full dose R‐CHOP in patients who are less than 80 or fit [140, 141], but appear better than palliation [142]. Although randomized trials do not exist comparing R‐CHOP to R‐mini‐CHOP, a recent propensity‐matched population‐based study in the Netherlands compared patients with a median age of 81. Two‐year PFS was 51% versus 68%, and OS was 60% versus 75% [140]. Alternative, unpublished, approaches may replace doxorubicin with gemcitabine or etoposide (R‐CEPP, R‐CDOP, R‐GCVP) [66]. However, a recent analysis of the FIL Elderly Project compared palliative versus curative intent therapies and found that patients 85 years and older who received an anthracycline, regardless of dose intensity, had better outcomes than those who did not receive an anthracycline [142]. Thus, higher dose intensity must be weighed against toxicity in elderly patients. G‐CSF prophylaxis is essential in this population to mitigate infection and prolonged cytopenias. Pretreatment with prednisone, that is “prephase treatment,” and/or rituximab should also be considered for elderly patients to improve performance status and potentially decrease toxicity [143, 144, 145]. The largest analysis to date failed to observe a survival benefit, but did support a decrease in chemotherapy delays [146].

Of note, among patients who received only palliative therapy, the use of rituximab was associated with improved 2‐year OS (42% vs. 22%) [142]. These data suggest that even frail or elderly patients ineligible for traditional standard of care full‐dose therapy may benefit from lymphoma‐directed therapy.

##### 4.2.4.2 Knowledge Gaps and Trials for Elderly/Frail Patients

One of the main difficulties in selecting the optimal regimen for elderly and/or frail patients stems from uncertainty regarding how best to assess frailty and how best to identify which patients will tolerate treatment poorly. There are many available tools, but nothing is consistently used in clinical practice [136].

Most clinical trials define young patients as adults up to and including age 65 years. The reality is that many patients who are between the ages of 65–80 and upwards may also tolerate full‐dose chemotherapy well, and chronological age alone poorly predicts how a patient tolerates lymphoma therapy [147]. This problem is further compounded because most tools are validated for use in cohorts treated with chemotherapy and not immunotherapy‐based approaches [147, 148, 149, 150]. Additionally, there are multiple validated tools for geriatric assessment in DLBCL, but there is no single tool that is consistently used to use to risk stratify these patients for trial eligibility and patients are not routinely stratified based on geriatric risk assessment. Furthermore, most studies in the elderly/frail population are single‐arm Phase 2 studies, which further complicates comparisons between approaches.

There are currently two main trial approaches under study in this population. One approach adds agents to an R‐mini‐CHOP backbone. The other approach is to omit chemotherapy entirely (Table 2).

Importantly, there are now randomized trials underway in this population: one is the SWOG1918 trial that is based on the observation that older patients with DLBCL have increased tumor methylation. This trial compares R‐mini‐CHOP with and without oral azacitadine [151]. Notably, it utilizes the FIL EPI tool for frailty assessment [148] to stratify patients based on fitness and frailty and also uses a serial comprehensive geriatric assessment (CGA) to assess the impact of therapy on functional status. Another randomized phase III trial investigates acalabrutinib in combination with R‐mini‐CHOP versus R‐mini‐CHOP alone (ARCHED/GLA 2022–1, NCT05820841) in patients over 80 or ages 61–80 who are unfit for R‐CHOP [152]. Finally, the POLAR BEAR trial (NCT04332822) is a randomized Nordic Lymphoma Group phase III trial comparing 6 cycles of R‐mini‐CHOP with polatuzumab‐R‐mini‐CHP in elderly patients ages 80+ or 75 years and frail as defined by a simplified Comprehensive Geriatric Assessment. The primary endpoint is PFS [153]. Several trials also examine CD20 x CD3 bispecific antibody combinations with R‐mini‐CHOP. Experience with epcoritamab‐R‐mini‐CHOP (EPCORE NHL‐2 trial) was reported at ASH 2025 [154]. Eligible patients were aged 75+ or ≥ 65years olds with comorbidities. They received 6 cycles of epcoritamab‐R‐mini‐CHOP followed by two additional cycles of epcoritamab. Although only 28 patients were treated, the CRR was 86% and two‐year estimated PFS and OS were 76% and 82%, respectively. Eighty‐five percent of patients achieved uMRD via ctDNA. The GLORY phase II trial [155] is a single institution, single arm study (NCT06765317) that will utilize glofitamab‐polatuzumab with a iPET2 response adapted approach to deescalate the number of cycles of pola‐R‐mini‐CHOP administered. After 2 cycles of glofitamab‐polatuzumab, if iPET2 is negative, patients will receive 4 cycles of glofit‐pola‐R‐mini‐CHOP. If iPET2 is positive without PD, patients will receive 6 cycles of glofit‐pola‐R‐mini‐CHOP.

There are also multiple trials underway that aim to omit chemotherapy entirely in the elderly, frail, or unfit DLBCL population. Many of these trials build upon the success of bispecific antibodies in the relapsed/refractory setting and utilize bispecific antibodies as the surrogate backbone for chemotherapy. Fixed duration subcutaneous mosunetuzumab for up to 1 year was studied via a Phase 2 trial in this patient population (NCT05207670) [156]. At 12 months follow‐up, CRR was 59% with a 69% PFS. Fixed duration epcoritamab for 1 year was also studied in this population in the EPCOR DLBCL‐3 trial with results strikingly similar to single agent mosunetuzumab; with 15 months follow‐up, ORR 70% with 58% CRR. 12‐month PFS was 54% and OS was 65% [157]. Finally, combination bispecific antibody trials in combination ADCs are preliminarily quite promising although longer‐term follow up is needed to determine the durability of these outcomes. The first results of the rituximab‐polatuzumab‐glofitamab (R‐pola‐glo) (NCT05798156) trial were presented at ASH 2025 [158]; this trial enrolled 80 patients who were unfit for full‐dose R‐CHOP. Patients received a steroid prephase followed by 6 cycles of R‐polatuzumab‐glofitamab and then 6 cycles of glofitamab consolidation. The median age was 80 years, about two‐thirds had intermediate‐high risk IPI, and 91% of patients were unfit or frail utilizing the simplified geriatric assessment (sGA). Eighty percent of patients were able to complete the entire regimen. At fifteen‐month follow‐up, 1‐year PFS and OS were 85% and 90%, respectively.

Additional chemotherapy‐free approaches focus on the use of frontline BTKi. A Phase 2 trial of zanubrutinib, rituximab, and lenalidomide (ZR2) enrolled 40 patients aged 75 years and older [159]. CRR was 65% with a 2‐year PFS and OS of 67% and 82%, respectively. Of interest, PFS and OS did not appear to be associated with traditional prognostic features, including COO, genetic subtype, DEL status, or IPI. However, an immunologically “active” tumor microenvironment was associated with responses; in particular, high tumor expression of HLA class I and II, as well as an increased number and activation of conventional type 1 dendritic cells were associated with response. A randomized trial of ZR2 versus R‐mini‐CHOP in unfit or frail older patients age 70+ with DLBCL is in progress (NCT05179733) [160]. There is also a phase II study of acalabrutinib + rituximab (ACRUE) for patients 80+ or those 65–79 and ineligible for chemoimmunotherapy (NCT05952024) [161]. Patients will receive up to 8 cycles of rituximab and 28 cycles of acalabrutinib. The primary endpoint is toxicity‐based.

## 5 Relapsed/Refractory DLBCL

Relapsed/refractory DLBCL remains a management challenge. Although patients may have up to two curative‐intent options in the relapsed/refractory setting (Figure 1), not all patients are optimal candidates for these options. Moreover, the available treatment options are time intensive, with a high risk for toxicity. In a recent population‐based study (US and Western Europe), approximately 32% of eligible patients did not receive second‐line DLBCL therapy, and in the third and later lines, 44% of patients were not treated [162]. If feasible, biopsy at each subsequent relapse is essential, especially prior to and after CAR‐T or bispecific antibodies due to the possibility of loss of target antigen, with multiple therapies that target CD19 and CD20, especially after bispecific antibody therapy (Figure 1) [163, 164, 165].

In general, CAR‐T and bispecific antibody‐based approaches are COO agnostic, whereas the other therapies, when response differs based on COO, are indicated.

### 5.1 Fit Patients, Curative Intent Options

#### 5.1.1 CAR‐T (Second Line+)

Curative intent therapy options for second line and beyond include anti‐CD19 chimeric antigen receptor T cells (CAR‐T) as well as salvage chemoimmunotherapy with autologous stem cell transplantation (ASCT) (Table 1). For fit patients with DLBCL that is primary refractory or relapsed within 12 months, anti‐CD19 CAR‐T remains the current standard of care (Figure 1). Currently approved CAR‐T target CD19, a pan B cell marker which is expressed on most lymphoma cells. These approvals for second line CAR‐T for LBCL were based on the ZUMA 7 [166, 167] and TRANSFORM [168] randomized phase 3 clinical trials (Table 1), which randomized ASCT‐eligible patients to CAR‐T versus salvage CIT + ASCT for chemosensitive disease. Both trials only enrolled transplant eligible patients, refractory to first line therapy or relapsed within 12 months. Both trials met their primary endpoints, with improved EFS in the CAR‐T arms. The ZUMA‐7 trial of axicabtagene ciloleucel demonstrated a survival benefit, with median survival not reached (estimated 4 year OS of 54.6%) at approximately 47.1 months' follow‐up in the arm that received axi‐cel, compared to 31.1 months (estimated OS 45%) in the ASCT arm [167]. Three‐year follow‐up for second line liso‐cel did not show an OS benefit (36‐month OS 63% vs. 52%), likely due to 66% of patients in the ASCT arm crossing over to the liso‐cel arm; however, it did confirm that most remissions appear to be durable with liso‐cel and most relapses occur within the first 6–12 months [169]; 4 year follow‐up presented at ASH 2025 also reported 52% PFS and 61.5% OS rates [170]. In addition to axicabtagene ciloleucel and lisocabtagene maraleucel, tisagenlecleucel is approved in the third line and greater setting for relapsed/refractory LBCL [171]; however, it is not approved for second line because the BELINDA study failed to show an EFS benefit over autologous stem cell transplantation (Table 1, Figure 1) [172]. Long‐term reported outcomes at 5 years and more with CAR‐T support the curative potential of anti‐CD19 for LBCL in approximately 30%–40% of patients [173, 174, 175]. If patients are autologous hematopoietic stem cell transplant ineligible, regardless of relapse timing after first line CIT, axi‐cel and liso‐cel are also standard of care in this setting based on the ALYCANTE and PILOT studies. Remissions were durable at 1 year and lasted a median of 2 years [176, 177].

#### 5.1.2 CAR‐T Resistance and Investigational Approaches

Mechanisms of resistance to CAR T‐cell immunotherapy can be largely classified into 3 main subgroups: (1) CAR T‐cell dysfunction secondary to exhausted or dysfunctional CAR‐T cells; (2) Tumor‐intrinsic resistance with antigen‐positive relapse or antigen‐negative relapse; and (3) Presence of an immunosuppressive or “cold” tumor microenvironment [178]. In an analysis of the French DESCAR‐T registry, 43.3% (238/550) experienced progression/relapse, with a median FU of 7.9 months [179]. For those with CART failure, 66% had progressive disease and 38.9% high LDH levels at the time of CART infusion. Failure after CAR T‐cell treatment occurred after a median of 2.7 months (0.2–21.5 months), and 22.7% presented with very early failure (day [D] 0‐D30); 42.9% had early failure (D31‐D90), and 34.5% had late (> D90) failure. For those with CART failure, the median PFS was 2.8 months and OS 5.2 months. Very early relapse and high LDH at the time of infusion were associated with worse PFS and OS. Only lenalidomide was associated with improved survival after CART failure, with an ORR of 11% (CR rate 6/7%) and 2nd PFS (PFS2) of 3.8 months, although this may represent selection bias rather than greater efficacy compared to other agents, including bispecifics.

The Peri‐CART US consortium reported outcomes on 514 patients across 13 centers treated with CAR‐T for B‐NHL between 2015 and 2021 [180]. A greater number of lines of therapy pre‐CAR‐T apheresis and bridging therapy were predictive of inferior progression‐free survival (PFS) and overall survival (OS). Similar to the DESCART data, median OS post‐CART failure was poor (5.5 months) and median PFS2 only 2.8 months. Refractory disease on day 30 had inferior OS (2.9 months), compared to 8 months in patients who had SD or PR on 1st post‐CART disease assessment. On univariate analysis, patients who received chemotherapy as 1 L treatment post–CAR‐T failure had inferior PFS2 compared to patients who received nonchemotherapy regimens (2.2 vs. 2.9 months; *p* = 0.047). In multivariate analysis comparing the top five first‐utilized regimens after CAR‐T, pola + BR or lenalidomide‐based regimens had better PFS2 (pola + BR HR, 0.097; 95% CI, 0.013–0.57; *p* = 0.01; lenalidomide‐based HR, 0.15; 95% CI, 0.026–0.76; *p* = 0.03, respectively). Allogeneic hematopoietic cell transplantation led to durable responses in over half of patients at 1 year.

In prospective studies that included patients who received prior CART therapies, bispecific antibody therapy [181, 182, 183, 184, 185, 186, 187, 188] (Table 3) and/or polatuzumab [189] achieved some notable responses. These observations, as well as reports of the ability of BsAb to trigger CAR‐T expansion [186], provide rationale for the currently ongoing S2114 study that uses consolidation with mosunetuzumab and/or polatuzumab vedotin for patients who achieve a PR or SD after CART cell therapy (NCT05633615) as well as the phase 2 ACCRU‐LY‐2201 study that randomizes patients with PR after CAR‐T to epcoritamab or observation (NCT06238648). Notably, ACCRU‐LY‐2201 excludes bulky disease (Table 2).

**TABLE 3:** Outcomes of bispecific antibody therapy for dlbcl relapsed/refractory after car‐t—select trials.

Bispecific antibody	Outcomes after prior CAR‐T	Outcomes for all patients	I & E criteria
Glofitamab B‐NHL^a^, ^b^	*N* = 52; 37% CRR; ORR 50% DOR—; DoCR 22 months (6.7‐NE)^b^	*N* = 155; 40% CRR; ORR 52%; PFS 4.9 months^a^ DOR 18.4months (13.7‐NR) ^1^; DoCR 26.9 months (16.8‐NR)^b^	≥ 4 weeks from CAR‐T^b^
Epcoritamab DLBCL^c^	*N* = 61; CRR 34%; ORR 54% DOR 9.7 months (5.4‐NR); DoCR NR	*N* = 157; 39% CRR; 63% ORR; DOR 12.0 months (6.6‐NR); DoCR 12.4 (12‐NR)	≥ 30 days from CAR‐T
Mosunetuzumab DLBCL^d^	*N* = 26; 12% CRR; 23% ORR^d^	*N* = 88; 24% CRR; 42% ORR; DOR 7.0 months (4.2‐NE); DoCR NR (9‐NE)	> 30 days from CAR‐T
Odronextamab NCT0229095 DLBCL^e^, ^f^	*N* = 60; 32% CRR; ORR 48% DOR 14.8 months (2.8‐NE), DoCR NR^e^	*N* = 127; 31.5% CRR; 52% ORR; DOR 10.2 months (5–17.9); DoCR 17.9 months (10.2‐NE)	Median 6.5 months (1.2‐46 months)^e^

Abbreviations: B‐NHL, B‐cell non‐Hodgkin lymphoma; CRR, complete response rate; DoCR, duration of complete response; DOR, duration of response; I & E, inclusion and exclusion; mo, months; NE, not evaluable; ORR, overall response rate; PFS, progression‐free survival.

^a^ Dickinson *NEJM* 2022.

^b^ Hutchings *ASH* 2023 #433. NCT03075696.

^c^ Thieblemont *J Clin Oncol* 2022; 41 (12): 2238–2247. NCT03625037.

^d^ Bartlett *Blood Adv* 2023; 7 (17): 4926–4935. NCT02500407.

^e^ Matasar *ASH* 2024 #866.

^f^ Kim Nat *Cancer* 2025; 6: 528–539. NCT03888105.

Alternative targets beyond CD19 and CD20 are needed to combat antigen‐negative lymphoma relapses. Although preliminary single institution phase I data were promising [190, 191], the multicenter phase 2 trial studying Stanford's anti‐CD22 CAR‐T was closed early due to a high incidence of IEC‐HS (immune effector cell hemophagocytic syndrome) and lower than expected duration of response likely due to changes in manufacturing and inclusion of patients whose lymphoma did not express CD22 [192]. A new study with the same anti‐CD22 CAR construct is enrolling. CAR‐T targeting BAFF‐R represents another promising, albeit early, approach [193]. Dual antigen‐targeting CAR‐T to prevent antigen‐negative relapse and potentially increase efficacy is another approach under study (targets have included CD19/CD20 and CD19/CD22) [194, 195, 196, 197, 198]. Although some incremental improvement has been reported, thus far, results have not indicated a major advance.

Fourth generation or armored CARTs are an approach to overcome an immunosuppressive tumor microenvironment. For instance, an anti‐CD19 armored CAR‐T secreting IL‐18 demonstrated the ability of a CAR‐T to achieve responses in patients whose lymphomas failed to respond to prior standard of care CAR‐T [199].

CAR‐T products comprised of less mature T cells have been associated with decreased exhaustion and improved outcomes. Improvements in manufacturing, such as shorter culture time [122] and CD62L+ enrichment [194], may improve the proportion of naïve and central memory T cells, resulting in increased efficacy. More data regarding product composition as well as long‐term follow‐up of these products are needed.

There are multiple methods to addressing CAR T‐cell dysfunction, including pharmacologic or nutritional manipulation, and the use of allogeneic CART, allogeneic CAR‐NK cells, and induced pluripotent stem cells (Table 2) [200, 201, 202, 203]. Furthermore, relapsed DLBCL is often rapidly progressive, and patients are unable to wait for manufacturing of the current autologous CAR‐T products. This highlights the need for the availability of either rapid manufacturing or ideally, “off‐the‐shelf” allogeneic products. Moreover, allogeneic products may result in healthier, less exhausted CAR‐T. A CRISPR‐edited allogeneic anti‐CD10 CART, vispacabtagene regedleucel (vispa‐cel; formerly CB‐010), addresses these issues and also has reported promising early efficacy of the Antler trial (NCT04637763) [204]. In addition to the efficacy, the toxicity profile is noteworthy for being very well‐tolerated. Mitigating toxicity is an essential step needed to broaden the usage of CAR‐T; the current toxicity profile of standard of care CAR‐T limits the settings in which these cellular therapies may be administered to highly specialized institutions.

#### 5.1.3 Autologous Stem Cell Transplantation

For fit patients who have relapsed DLBCL after more than 12 months and are transplant eligible, salvage platinum‐containing chemoimmunotherapy with R‐ICE (rituximab, ifosphamide, carboplatin, etoposide), R‐DHA (rituximab, dexamethasone, cytarabine) plus either cisplatin, (R‐DHAP), carboplatin (R‐DHAC) or oxaliplatin (R‐DHAX), or R‐GDP (rituximab, gemcitabine, dexamethasone, carboplatin/cisplatin/oxaliplatin) followed by ASCT for chemosensitive disease, including those patients with partial responses, remains the standard second‐line curative intent approach (Figure 1, Table 1) [205, 206, 207, 208].

### 5.2 Patients Ineligible for or Relapsed After CAR‐T

#### 5.2.1 Bispecific Antibody Monotherapy (Third Line)

The CD20 x CD3 bispecific antibodies, epcoritamab and glofitamab, were approved in the United States for third line and later therapy of DLBCL in 2023 (Figure 1, Table 1) [123, 181, 209, 210]. Glofitamab is administered intravenously for up to 1 year of treatment. Epcoritamab is administered subcutaneously and given as continuous dosing until progression or toxicity. Aside from cytokine release syndrome with initiation and step up dosing of these BsAb, the major toxicity of these drugs remains infection. Risk of infection appears related to total duration of BsAb exposure regardless of indefinite or time‐limited duration [211, 212]. Seventeen percent of patients discontinued epcoritamab due to AEs and a quarter of patients had a grade 3+ infections [211], whereas 3% of patients discontinued glofitamab due to AEs and 15% of patients receiving glofitamab had grade 3+ infections. Although the median PFS was only 4–5 months in these heavily pretreated patients with DLBCL, CR rates are similar, around 40% for both trials, and the median duration of CR was 30 months for glofitamab [212] and 38 months for epcoritamab (Table 3) [213]. Of note, after 4 years of follow‐up, only 18 of the original 157 patients were continuing to receive epcoritamab; the mean duration of epcoritamab received was 12 months [213]. A large dataset describing real world outcomes of 245 patients treated at academic centers with epcoritamab or glofitamab was recently reported [214]; efficacy was lower than reported in clinical trials: ORR was similar (52%) but CRR was only 25%, PFS was 2.3 months (95% CI 1.9–3.7), and OS was 7.8 months (95% CI: 6.1–11). The poorer efficacy was likely a reflection of treating higher‐risk patients than those enrolled in the registrational trials given most would have been ineligible for these trials. Unsurprisingly, but important to note, baseline CD20 expression was associated with longer PFS compared to lack of CD20 expression, again underscoring the importance of baseline biopsy to assess for target antigen expression for patients planned for BsAb. Follow‐up was only 6 months, but CRs appeared durable, consistent with long duration of CRs (DoCRs) reported by clinical trials; however, longer follow‐up is needed to confirm this. Finally, grade 3+ CRS appeared higher in patients who received epcoritamab, but the significance of that is unclear.

Trials of bispecific antibody therapy utilizing other targets are needed due to the significant proportion of patients with target antigen negative lymphoma relapses after CD20 × CD3 BsAb. For example, AZD0486 is a CD19 × CD3 BsAb that is being studied in a phase I clinical trial (NCT04594642). In a heavily pretreated DLBCL cohort, 33% of patients had a CR and 29% of patients who had received prior CAR‐T had a CR. CRS was mild (only grade 1 in a third of patients) although ICANS occurred in 27% [215]. Other targets under study include BAFF‐R × CD3 BsAb in development [216] (Table 2).

#### 5.2.2 Bispecific Antibody Combinations (Second Line and Later)

One of the drawbacks of bispecific antibody monotherapy is the need for ramp‐up (“step‐up dosing”) to mitigate CRS related to the initiation of bispecific antibodies. This ramp‐up period typically lasts about 2–3 weeks until the therapeutic dose is administered and presents challenges in the setting of rapidly progressive disease. Thus, bispecific antibodies in combination with chemotherapy and/or ADCs are an attractive combination to administer immediate disease control.

Moreover, they appear to have less toxicity and are more readily available than the approved CAR‐T products (Table 1, Figure 1).

##### 5.2.2.1 Bispecific Antibodies and Chemotherapy

Bispecific antibodies in combination with salvage chemotherapy have been found more efficacious than salvage chemotherapy with rituximab. The phase 3 Starglo trial randomized 274 patients 2:1 to receive either second‐line glofitamab‐gemcitabine/oxaliplatin (glofit‐gem/ox) or rituximab‐gemcitabine/oxaliplatin (R‐gem/ox) [217]. Patients received 12 cycles of glofit and 8 cycles of gem‐ox every 3 weeks. At a median follow‐up of 11.3 months, this trial met its primary endpoint of improvement in overall survival; median OS was 25.5 months in the glofit‐gem/ox arm versus 12.9 months in the R‐gem/ox arm. Similarly, median PFS with glofit‐gem/ox was longer (13.9 vs. 3.6 months). These results led to approval of this combination in the EU; however, this approach is not FDA approved in the US. Review of the Oncologic Drugs Advisory Committee (ODAC) Meeting [218] showed concern that 50% of the Starglo population was Asian with only a very small group of North American patients (*N* = 25). With the caveat of inadequate power and overlapping 95% CI, the small group of US patients fared numerically worse in the glofit‐gem/ox group than R‐gem/ox (OS: 13.3 months vs. NR, PFS: 27.1 vs. 7.5 months). Unlike the small US cohort, the European cohort showed consistent trend towards improved OS and PFS with glofit‐gem/ox similar to the full cohort (OS: 21.2 months vs. 13.1 months) and was granted EU approval.

Epcoritamab‐gem/ox was also studied after 2 or more prior therapies in patients who were transplant ineligible in the EPCORE‐NHL 2 trial [219]. This was a single arm trial that enrolled 103 patients. The primary endpoint of a 15% improvement in ORR from 46% was met with an observed ORR of 85%. With a median follow‐up of 13.2 months, median PFS was 11.2 months (8–14.7 months) [219]; 2 year follow‐up presented at ASH 2025 showed approximately 48% of patients remained progression‐free [220]. Major differences in these trial designs included indefinite epcoritamab versus fixed duration glofitamab for 1 year, and different frequencies of gem/ox administration (2 weeks in EPCORE NHL 2 vs. 3 weeks in Starglo). Of note, both glofitamab and epcoritamab are second line and later options on the NCCN guidelines for relapses more than 12 months after initial therapy in transplant ineligible patients.

#### 5.2.3 Bispecific Antibodies and ADCs

Bispecific antibodies in combination with antibody‐drug conjugates are another compelling option for management of relapsed DLBCL. Importantly, bispecific antibodies in combination with ADCs have now demonstrated efficacy over standard of care chemotherapy.

Mosunetuzumab‐polatuzumab (mosun‐pola) was initially studied in a Phase Ib/II study (NCT03671018), which showed the combination was feasible with durable responses [187]. An analysis of a Phase II randomized cohort for patients who had received at least 1 prior therapy compared fixed duration mosun (SC administration)‐pola versus R‐pola was reported at ASH 2024 [221]. Mosunetuzumab was given for cycles 1–8 with SUD during Cycle 1. Polatuzumab was given D1 of Cycles 1–6. Rituxmab was given on D1 of Cycles 1–8. The primary endpoint was BORR. ORR was 78% (vs 50%) with 58% (vs 35%) CRR. Median follow‐up was 18 months, with median PFS NR versus 6.4 months and DOR was NR versus 10.1 months. CRS occurred in 10% (4/40 pts) receiving mosun‐pola. Serious infections occurred in 23% of patients and 18% of patients receiving R‐pola. Treatment was discontinued due to AEs in 3 patients (8%) due to peripheral neuropathy and COVID PNA, whereas 2 pts on R‐pola discontinued due to PN and extremity pain.

The SUNMO trial (Table 1) randomized 208 patients with relapsed/refractory LBCL ineligible for autologous stem cell transplantation to subcutaneous mosunetuzumab‐polatuzumab (mosun‐pola) or rituximab‐gemcitabine, and oxaliplatin (R‐GemOx) [188]. With a median follow‐up of 23 months, the trial met its primary endpoints of improved ORR and PFS. ORR was significantly greater with mosun‐pola (70% vs. 40%) and PFS was significantly longer with mosun‐pola (11.5 months [95% CI: 5.6–18 months] versus 3.8 months [95% CI: 2.9–4.1 months]) compared to R‐GemOx. There was a low rate of CRS (26% all grades, typically only fever and less than 5% of CRS was Grade 2–3). CRS predominantly occurred during cycle 1. Grade 3 neutropenia was similar, however, grade 3+ thrombocytopenia and anemia were less common with mosun‐pola, and peirpheral neuropathy was less frequent (24% vs. 42%). Importantly, there were more infections of any grade in the mosun‐pola arm (51% vs. 31%) and COVID‐19 was also more common in the mosun‐pola arm (15% vs. 3.1%). Few patients received prior CAR‐T, thus outcomes in this specific, very high‐risk cohort, are unavailable. This trial establishes mosun‐pola, a bispecfic antibody‐ADC combination, as superior to R‐GemOx and a new standard of care in the relapsed/refractory setting.

Of note, a phase Ib/II trial of glofitamab‐polatuzumab was recently reported [222]. Twenty‐two percent of patients received prior CAR‐T. ORR was 78% with a CRR of 60%. With a median follow‐up of 33 months, median PFS was 12.3 months and median OS was 34 months. CRS was observed in 43% of patients; the majority were grade 1–2. Nine percent of patients had grade 5 AEs and 15% discontinued therapy due to AEs. With the caveats of cross trial comparisons, the toxicity of this regimen appears higher than with mosun‐pola, with comparable efficacy.

Preliminarily, other ADCs appear to have similar synergy with bispecific antibodies. The LOTIS‐7 trial (NCT04970901) evaluates loncastuximab combinations for B‐cell NHL. Of particular interest is the cohort evaluating loncastuximab + glofitamab. Glofitamab is administered per standard of care. Loncastuximab is administered every 21 days with glofitamab for a total of 8 cycles. Patients then receive an additional 4 cycles of glofitamab monotherapy. The primary endpoint is safety. Preliminary results of 41 patients with LBCL were presented in 2025 [223]. Toxicity was manageable (39% CRS, 2.5% Grade 3, without Grade 4–5 CRS and no severe ICANS) and known loncastuximab AEs did not appear increased. Notably, the ORR was 93% with an 87% CRR.

In summary, based on the described ORR and PFS with bispecific antibody + ADC combinations, this approach appears highly active. Although we lack a direct comparison, this treatment strategy likely adds additional efficacy over bispecific antibody‐chemotherapy combinations or bispecific antibodies alone. Longer follow‐up is needed to clarify the durability of bispecific antibody‐ADC remissions. Trials of bispecific antibodies are reviewed in Table 3.

#### 5.2.4 Sequencing of CAR‐T and Bispecific Antibodies

The optimal sequencing of bispecific antibody± ADC versus CAR‐T is currently unknown, but selection may be influenced by practicality, including access to a center that specializes in CAR‐T, tumor antigen expression, and how rapidly DLBCL is progressing.

Outcomes of bispecific antibody monotherapy after CAR‐T are better than other standard of care options but remain suboptimal with only about 30% of patients achieving CR (Table 3). This CR rate may also be an overestimate because trials of bispecific antibodies required a washout of at least 1 month post‐CAR‐T infusion (Table 3). In general, patients who received BsAb within close proximity to CAR‐T had poorer outcomes [186, 214, 224], especially within the first 3–9 months. This trial experience is also now confirmed in published real‐world experiences [214, 224]. Early relapse within 3 months after CAR‐T infusion had a 10% CRR and a 2.2 month median PFS, whereas relapses 4–6 months after CAR‐T had a 25% CRR and a 3.7 month median PFS, and late relapses at least 6 months after CAR‐T had a 45% CRR with a 10.5 month PFS [224]. Another real world analysis reported on 19 patients with paired biopsies before and after BsAb. In a different real world series, 17 of 19 (90%) patients' tumors lost CD20 expression by either flow cytometry or IHC at progression [214]. This has important implications for sequencing, especially if bispecific antibodies move to earlier lines of therapy in the future.

Due to the potential use of bispecific antibody therapy in earlier lines of therapy, outcomes of bispecific antibodies followed by CAR‐T are also important. One of the DESCAR‐T registry analyses reported a small analysis of patients with relapsed/refractory LBCL treated with CD19‐targeted CAR T cells after prior CD20 × CD3 bispecific antibody therapy [225]. Time from last bispecific antibody dose to CAR‐T infusion was around 3 months (range: 47–572 days). Estimated 1 years PFS was 42% and OS was 55%. They compared these outcomes to a bispecific antibody‐naive control group using propensity score matching. This control group had similar 1‐year PFS and OS as well as toxicity, suggesting that CAR‐T is effective after prior BsAb exposure if each targets different antigens. Lack of response to BsAb was not associated with lower CAR‐T ORR.

In our practice, CAR‐T remains the preferred second‐line approach due to demonstrated long‐term durable responses (Figure 1). However, bispecific antibody‐ADC combinations are an important addition to therapeutic options for LBCL. Their immediate availability makes it more feasible to administer in more practice settings compared to CAR‐T and also makes them a more attractive option for rapidly progressive DLBCL.

#### 5.2.5 Antibody‐Drug Conjugates

Polatuzumab Vedotin is a humanized anti‐CD79b monoclonal antibody also conjugated to MMAE. Its approval was based on GO29365 (Table 1), a phase 2 trial that randomized 80 transplant‐ineligible relapsed/refractory DLBCL patients after a least one prior line of therapy to either polatuzumab‐vedotin with rituximab‐bendamustine (BR) or BR alone. At a median follow‐up of 27 months, the study reported an ORR rate of 45% in Pola‐BR versus 17.5% in the BR arm alone, with a CR of 40% versus 15% and a median DOR of 12.6 months versus 7.7 months [226]. Four‐year survival follow‐up confirmed both a PFS and OS benefit in the pola‐BR arm [227].

The POLARGO trial (NCT04182204) [228] is a randomized phase III study that compared up to 8 cycles of polatuzumab‐R‐GemOx to R‐GemOx alone (Table 1). Patients were eligible if they had DLBCL, were transplant ineligible, and had received at least one prior therapy. The study randomized 250 patients and met its primary endpoint; OS after pola‐R‐GemOx was 19.5 months (95% CI 13‐NE) versus R‐GemOx 12.5 months (95 CI: 8; 9–15.8). Tumors underwent GEP, and both GC and ABC‐DLBCL experienced a benefit. Of note, AEs were higher as well with pola‐R‐GemOx: thrombocytopenia, infections, and peripheral neuropathy. Treatment discontinuation rates were higher and there were more grade 5 AEs (due to infections) with pola‐R‐GemOx (23% versus 8%) than R‐GemOx. Thus, polatuzumab‐R‐GemOx or polatuzumab/mosunetuzumab are the most active available polatuzumab combinations in the relapsed/refractory setting. These are potential options for patients with relapsed/refractory DLBCL who are CAR‐T ineligible (second line and later) or who have relapsed after CAR‐T (third line and later). The impact of frontline pola‐RCHP on responses to later polatuzumab‐containing regimens is unknown.

Loncastuximab Tesirine (ADCT‐402) is a humanized anti‐CD19 monoclonal antibody conjugated to a pyrrolobenzodiazepine dimer (PBD) toxin. Loncastuximab is FDA approved for third‐line and greater therapy of relapsed/refractory DLBCL based on the results of the LOTIS‐2 trial [229, 230]. This trial (Table 1) was a single arm Phase 2 study (NCT03589469) in 183 patients with RR B‐NHL who had failed two or more prior therapies. Outcomes were reported for 138 evaluable DLBCL patients with an ORR of 42.3% and a median DOR of 4.5 months. Notably, the ORR in DLBCL patients 75 years and older, with primary refractory disease or DHL/THL was 55.6%, 23.3%, and 21.7%, respectively. The LOTIS‐5 trial (NCT04384484) is a phase 3 randomized study of loncastuximab with rituximab versus R‐GemOx for patients with r/r DLBCL after at least 1 prior therapy who are transplant ineligible; it has completed enrollment and the primary endpoint is PFS [231].

Finally, the combination of brentuximab vedotin, lenalidomide, and rituximab (BV + len + R) was approved in February 2025 for patients with third line or greater relapsed/refractory DLBCL who are ineligible for hematopoietic SCT or CAR‐T (Table 1). ECHELON‐3 [232] was a randomized, double‐blind, placebo‐controlled phase 3 trial that compared lenalidomide (len) + rituximab (R) with and without brentuximab vedotin (BV). Median follow‐up was 16 months, and OS and PFS were significantly longer in the BV + len + R arm compared to placebo + len + R (OS: 13.8 months versus 8.5 months, and PFS: 4.2 months versus 2.6 months, respectively). Notably, responses were observed regardless of COO or CD30 expression. Although this is now an approved regimen, the median PFS with this regimen was quite short, so we typically sequence this approach after CAR‐T and bispecific antibody combinations.

#### 5.2.6 Tafasitamab‐Lenalidomide

Tafasitamab‐lenalidomide (tafa‐len) is available second line and later if not a candidate for transplant. Notably, transplant‐ineligible patients may nevertheless be CAR‐T eligible if sufficiently fit. The L‐MIND study (Table 1) was a phase 2 study in patients with R/R DLBCL ineligible for autologous stem cell transplant after 1–3 prior therapies who received intravenous tafasitamab, a humanized anti‐CD19 monoclonal antibody with modified Fc to enhance antibody‐dependent cellular cytotoxicity (ADCC) and cell‐mediated phagocytosis, in combination with oral lenalidomide for 12 cycles followed by tafasitamab monotherapy until progression [233]. The primary endpoint was ORR. After a median follow up of 13.2 months, 48 (60%; 95% CI 48–71) of 80 patients who received tafa‐len had an objective response and 34 (43%; 32–54) had a complete response. The median time to first response was 2 months (IQR 0.4–12.6). Neutropenia was the most common grade 3 or higher toxicity and occurred in 48% of patients; notably, 51% of patients had a serious adverse event and 25% discontinued treatment with one or both drugs due to adverse events. Results from the five‐year follow‐up showed a median PFS of 11.6 months. ORR was 57.5% and CRR was 41.3%; patients who had received 2–3 prior therapies had lower response rates and PFS: 47.5% ORR, 30% CRR, median PFS 7.6 months. Irrespective of number of prior therapies, median DOR was not reached [234]. Also of interest, relapse more than 12 months after frontline therapy was associated with higher ORR versus primary refractory or relapse < 12 months: 85% versus 50%. Finally, non GCB phenotype had a higher ORR (68%) compared to GCB (47%), but a large portion (25%) could not be classified, which makes it difficult to dissect the impact of COO on response. The combination tafa‐len was FDA approved in 2020 for the treatment of transplant ineligible patients with DLBCL; however, optimizing patient selection may be critical to replicate responses observed on L‐MIND. In particular, a large series of real world outcomes of tafa‐len reported 19% ORR in CAR‐T exposed patients, versus 36% ORR in CAR‐T naïve patients; PFS was similarly short with prior CAR T (1.7 vs. 2.8 months) [235]. These poorer outcomes may have been impacted by a more heavily pre‐treated real world population.

Of note, the potential for tumor loss of CD19 after tafasitamab has not been well studied, but limited series suggest that this is not as common an occurrence as with the CD20 × CD3 BsAb. In particular, lab studies suggest that “loss” of CD19 after tafasitamab is due to “masking” of CD19 by tafasitamab rather than true loss of antigen [236]. This hypothesis is supported by findings of some small series describing biopsies obtained after tafasitamab which found that biopsies obtained more than 3–4 weeks after tafasitamab were CD19‐positive, whereas biopsies obtained in less than 3–4 weeks after tafasitamab were negative [235, 237, 238]. Nevertheless, caution should be exercised in using tafasitamab prior to CAR‐T until we have more definitive data to reassure that loss of antigen is not an issue after tafasitamab or that tafasitamab does not adversely impact CAR‐T.

### 5.3 COO‐Dependent Therapies

#### 5.3.1 Selinexor

Selinexor is an oral, reversible exportin 1 (XPO1) inhibitor that was FDA approved in 2020 for patients with relapsed/refractory DLBCL after 2 or more prior therapies. The approval was based on the results of the international Phase 2 SADAL trial which enrolled patients with transplant ineligible or transplant refractory R/R DLBCL with an ECOG 0–2 after at least 2 prior lines of therapy. Patients received selinexor on days 1 and 3 weekly until progression or toxicity. The primary outcome of ORR was achieved in 28% of patients (12% CR, 17% PR) with a median duration of response of 9.3 months. Median progression‐free survival was 2.6 months. Responses to selinexor were best in those patients with GCB DLBCL (34% vs. 21% ORR, 14% vs. 10% CR) and those patients with c‐myc IHC expression less than 40% (ORR 13% vs. 42%). While selinexor has a novel mechanism and offers a convenient route of administration, Grade 3–4 hematologic toxicity occurred in almost all patients (92%,117/127) [239].

Selinexor has also been studied in R/R DLBCL with TP53 aberrations in combination with salvage chemotherapy with in vitro analyses suggesting a synergistic effect of selinexor with cisplatin sensitivity in this population [240].

The role of selinexor remains to be defined given its toxicity profile and the advent of other effective treatments in the R/R setting.

#### 5.3.2 ViPOR (Venetoclax, Ibrutinib, Prednisone, Obinutuzumab, Lenalidomide)

This regimen is not widely available as standard of care but demonstrated the ability of a time‐limited non‐chemotherapy regimen to induce long‐term durable remissions and possibly cures without using a CAR‐T or transplantation approach.

This regimen was developed with the hypothesis that simultaneously targeting multiple cancer survival pathways would be more effective: ibrutinib, B‐cell receptor (BCR)‐dependent NF‐kappaB activation; glucocorticosteroids, BCR signaling; venetoclax, BCL2 inhibition; lenalidomide, Ikaros/Aiolos, indirectly IRF4; obinutuzumab used for innate immune responses to malignant B cells. This single‐institution, phase Ib/II trial demonstrated proof of concept that fixed duration, non‐chemotherapy regimen could induce durable remissions in the relapsed/refractory DLBCL population [241]. Patients with DLBCL who had received prior anthracyclines were eligible. ViPOR was administered (ibrutinib D1‐14, prednisone D1‐7, obinutuzumab D1‐2, lenalidomide D1‐14) every 21 days for 6 cycles until PD or toxicity. Of 60 patients enrolled, 50 had DLBCL. Forty percent of patients had prior CAR‐T and 58% had refractory DLBCL. ORR was 54% and CRR was 38%. Notably, CRs were only observed in patients with non‐GCB DLBCL and HGBCL, in particular GCB tumors with MYC and BCL2 rearrangements. Median follow‐up was 40 months and 2‐year PFS was 34% for all patients with DLBCL; at 2‐years, 78% of CRs remained durable. In patients who had received prior CAR‐T, PFS was 30% at 2 years. Therapy was reduced in 17% and delayed in 25%. Grade 3–4 thrombocytopenia occurred in 45%, anemia in 25%, neutropenia in 55%, and hypokalemia in 29%. All grade diarrhea occurred in 68%. ViPOR(P), with the addition of polatuzumab, has also been shown feasible without additional toxicity in a single center study [242]. Median follow‐up of 28 months showed 2‐year PFS 49% in non‐GCB DLBCL and 67% in ABC DLBCL by GEP. This trial is ongoing. The ViPOR regimen is currently under study (EA4231/NCT06649812) in a multicenter phase II trial enrolling CD10 negative DLBCL or HGBCL with MYC and BCL2 rearrangements.

Other targeted degraders and immunomodulatory combinations with preliminary data in relapsed/refractory DLBCL include BCL6 degraders (NCT06090539) [243], golcadomide with rituximab (NCT03930953) [244], and zanubrutinib + lenalidomide (NCT04436107) [245] (Table 2).

## 6 Conclusions

The armamentarium for DLBCL has rapidly expanded in the past few years, offering new therapies for both untreated and relapsed/refractory DLBCL that are improving patient survival. With this plethora of new treatment approaches come new questions and increasing complexity in the risk stratification and treatment of DLBCL. Optimizing how best to incorporate immunotherapy into earlier lines of therapy, and for whom, remains to be established. Approaches to mitigate toxicity are another unmet need, especially for frail patients. New approaches to risk stratify and monitor patients in this evolving therapeutic landscape, assist with patient‐specific therapy selection, and optimize treatment sequencing are sorely needed. Finally, our growing biologic understanding of DLBCL and new immunotherapies have yet to consistently be translated into approaches that are routinely available in most practices outside of a tertiary care center or clinical trial. Recent advancements in understanding the diversity of DLBCL and treatment options have been tremendous and the future for curing ever more patients with DLBCL looks bright, but there remains much work to be done.

## Funding

This work was supported by the Lymphoma Research Foundation. Center for Drug Evaluation and Research, FDA (1R01FD008168‐01A1). National Cancer Institute (NCI P30 CA016520).

## Ethics Statement

The authors have nothing to report.

## Consent

The authors have nothing to report.

## Conflicts of Interest

E.A.C. reports research funding from Genmab, AbbVie, AstraZeneca, Nurix, CARGO and advisory board participation for Genmab, AbbVie, AstraZeneca, Genentech. E.B.T. reports research funding from AstraZeneca and advisory board participation for AstraZeneca, AbbVie, Bristol Myers Squibb, Genentech, and Sanofi. S.K.B. reports research funding from Vittoria Biotherapeutics, and advisory board participation for Acrotech, Citius, Daiichi Sankyo, Kyowa Kirin, ONO, and SecuraBio; DSMB for Janssen.
